# Robust Photoelectric Biomolecular Switch at a Microcavity-Supported Lipid Bilayer

**DOI:** 10.1021/acsami.1c06798

**Published:** 2021-06-14

**Authors:** Guilherme B. Berselli, Aurélien V. Gimenez, Alexandra O’Connor, Tia E. Keyes

**Affiliations:** School of Chemical Sciences, National Centre for Sensor Research, Dublin City University, Dublin D09 FW22, Ireland

**Keywords:** photo-device, protein photoactivation, bacteriorhodopsin (bR), microcavity-supported lipid bilayer (MSLB), bioinspired electrical device

## Abstract

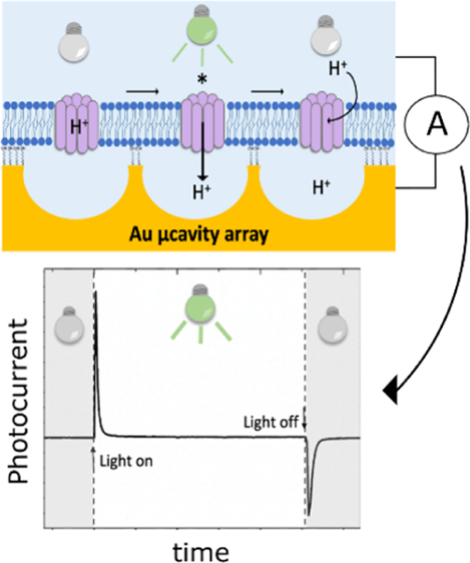

Biomolecular devices based on photo-responsive proteins have been widely proposed for medical, electrical, and energy storage and production applications. Also, bacteriorhodopsin (bR) has been extensively applied in such prospective devices as a robust photo addressable proton pump. As it is a membrane protein, in principle, it should function most efficiently when reconstituted into a fully fluid lipid bilayer, but in many model membranes, lateral fluidity of the membrane and protein is sacrificed for electrochemical addressability because of the need for an electroactive surface. Here, we reported a biomolecular photoactive device based on light-activated proton pump, bR, reconstituted into highly fluidic microcavity-supported lipid bilayers (MSLBs) on functionalized gold and polydimethylsiloxane cavity array substrates. The integrity of reconstituted bR at the MSLBs along with the lipid bilayer formation was evaluated by fluorescence lifetime correlation spectroscopy, yielding a protein lateral diffusion coefficient that was dependent on the bR concentration and consistent with the Saffman–Delbrück model. The photoelectrical properties of bR-MSLBs were evaluated from the photocurrent signal generated by bR under continuous and transient light illumination. The optimal conditions for a self-sustaining photoelectrical switch were determined in terms of protein concentration, pH, and light switch frequency of activation. Overall, a significant increase in the transient current was observed for lipid bilayers containing approximately 0.3 mol % bR with a measured photo-current of 250 nA/cm^2^. These results demonstrate that the platforms provide an appropriate lipid environment to support the proton pump, enabling its efficient operation. The bR-reconstituted MSLB model serves both as a platform to study the protein in a highly addressable biomimetic environment and as a demonstration of reconstitution of seven-helix receptors into MSLBs, opening the prospect of reconstitution of related membrane proteins including G-protein-coupled receptors on these versatile biomimetic substrates.

## Introduction

Molecular machines, capable of reversible molecular motion or vectorial charge transport in response to optical or electrochemical stimuli, are widely proposed as constituents of Boolean logic gates for high density data storage and processing or for signal processing in imaging and sensing. As recently discussed by Leigh et al., there are two approaches generally taken in design of molecular machines, technomimetic or biomimetic, where the structures are inspired by real-world mechanical machines in the former and by molecular biological switches and signals in the latter.^1^ The advantage of biomimetics is that such systems are already molecular based, and biology offers the advantage of iteratively evolved structures and mechanisms to produce nanomechanical devices that offer robust addressablility by light or changes to potential. Such biological molecular machines include functions such as ATP synthase, neural transport, force generation, cell motility and division, membrane channels, and ions pumps. All offer opportunities for the development of smart switchable materials.^2^ Also, the manipulation of such biological structures in engineered self-assembled biomolecular devices is emerging as a domain with extensive potential for advances in nanoengineering, material science, as well as an avenue to new biological insights.^3−5^

However, the majority of biological molecular machines are membrane proteins, thus their structure and conformation, which are intrinsically linked to their efficient operation, rely on their proper insertion or orientation at the lipid membrane. Thus, the reconstitution of such biomaterials into bioengineered structures that closely mimic the key elements of the biological membrane is crucial to achieving optimal and stable response.

In biology, signal transduction and cell signaling frequently rely on biomolecular switches such as protein conformational change to create membrane proton gradients, instigate processes such as protein aggregation, and cellular viral entry.^6,7^ Many applications of biomolecular machines in artificial devices particularly rely on the ability of biomaterials to respond electrically under light irradiation. Among these, particular attention has been paid to biohybrid engineered devices focused on self-assembled protein-based photonic devices, such ion channel and transporter proteins in artificial membranes.^8,9^ Such retro-bioengineering of photochromic biological machines are finding applications in photochemical cells, biosensors, solar fuel, and energy storage/conversion.^10^

Bacteriorhodopsin (bR) is one of the most studied biological machines in this regard. It is a photo-initiated proton pump of the *Halobacterium salinarum* membrane. bR converts solar into chemical energy by pumping protons against a proton gradient from the cytosolic to the extracellular side of the cell membrane. The bR photocycle, which completes in about 10 ms at room temperature, occurs through a series of seven spectroscopically distinguishable steps bR → K → L → M1 → M2 → N → O → bR. The first is initiated by the photoinduced all-trans-to-cis isomerization at C13=C14 of the retinal, culminating in proton transfer across the bacterial membrane.^11,12^ Because of its relatively robust structure, bR has been studied with demonstrable retention of photoactivity, outside of its native membrane environment in thin solid films. Therefore, bR is an attractive model for hybrid bioelectronic devices,^13^ across diverse applications including optical memories, it is used in photovoltaic cells, artificial cells, and artificial retina prostheses.^10,14−16^

However, like most biological machines, bR is a membrane protein. It is a seven-pass integral protein, meaning that, in nature, it is integrated into the cell membrane of the organism through seven topogenic α-helices that also encase the photoactive retinal trigger. The membrane environment is thus intrinsic to conformation and function. For bR, as the retinal is encapsulated within the membrane-bound helices in its native environment, its coupling to these helices and the conformational changes that retinal instigates occur within the membrane and therefore the dynamics are likely evolutionarily optimized for operation in this native environment and, while bR is remarkably robust compared to other membrane proteins, to the extent that many hybrid applications have not required the protein to be embedded in its native environment, there are significant advantages to bR integration into functional hybrid devices that encompass the lipidic environment and intra- and extracellular analogues. Not least because using a membrane enables use of wild-type and thus inexpensive bR directly without the need for genetic modification.^10^ Furthermore, given the complexity of the photocycle, ensuring conformational integrity of the protein should ensure closest biomimicry so that the evolutionarily iterated dynamics of the photoswitch apply. Indeed, distinct changes to the dynamics of the photocycle are observed depending on the protein microenvironment.^17^ In addition, stable, interfacial hybrid devices that incorporate integral proteins into true, fluidic lipid membranes, while rare, offer opportunities to diversify from robust proteins such as bR to other less studied membrane proteins, broadening potential access to a wide range of molecular machines.^18,19^

Ideally, a lipid/protein (L/P)-based artificial model must exhibit controllable lipid composition, good addressability, by interfacial (electrochemical) and spectroscopic methods, and crucially, maintain lipid membrane lateral fluidity, avoiding frictional interactions with the underlying substrate and also potentially protein denaturing surface interactions.^20,21^ This combination of properties is not trivial to accomplish. Solid-supported lipid membranes and associated cushioned/tethered variants are robust and stable lipid models with excellent addressability and are therefore widely used as artificial membrane models. For bR where the solid support is conducting, the interface provides a means to study the photocurrent.^22,23^ However, reconstituted membrane proteins rarely show mobility. Liposomes and more recently lipid nanodisks are excellent platforms for ensuring a native like environment for reconstituted protein but suffer issues with addressability. However, solid-supported lipid membranes have some fundamental drawbacks, for example, they typically exhibit reduced lipid lateral mobility and the membrane-substrate distance is usually not sufficiently large to avoid direct contact between transmembrane proteins.

To avoid substrate–membrane interactions, new and more biomimetic artificial lipid models based on pore-suspended lipid membranes have been proposed, which combine the versatility of substrate-supported lipid bilayers with the membrane fluidity observed with freestanding membranes. For instance, the photoelectrical response of bR has been studied in black lipid membranes,^19^ nanopore-supported lipid bilayers,^24^ and nanodiscs.^25,26^ Such approaches ensure that the membrane is not in contact with a solid interface. A potentially useful lipid-based device in this regard for molecular machines is the microcavity-supported lipid bilayer (MSLB), which combines the membrane fluidity observed in free-liposomes with the versatility and addressability of supported lipid bilayers. The pore-suspended character of MSLBs in contrast to classical SLBs ensures that there is bulk aqueous environment at both interfaces of the bilayer. The deep aqueous reservoir of the well supporting the suspended bilayer ensures reconstituted membrane proteins attain full lateral mobility. Although single pass proteins, including integrin and glycophorin, have been reconstituted into MSLBs,^27^ multi-pass proteins have not been reconstituted till date. Thus, also light-addressable ion channel proteins such as bR to artificial membranes spanned over microdimensioned cavity arrays, and their electrical activity was not yet explored. Reconstitution of proteins into such devices is of value because they combine the qualities of true, compositionally versatile lipid bilayer with fluidity, including the reconstituted protein and optical and electrochemical addressability.

Herein, we report on a method of reconstitution of bR at an MSLB using a hybrid two-step method involving fusion of proteoliposomes containing bR to pre-deposited lipid monolayers spanned over aqueous-filled microcavity arrays. This method could reliably be used to reconstitute different densities of bR to the MSLB permitting investigation of the biophysical properties of formed artificial membranes and the photoelectrical activity of bR as a function of the concentration. The lipid bilayer formation and reconstituted bR at MSLBs were evaluated using fluorescence lifetime cross correlation spectroscopy (FLCCS) and fluorescence lifetime correlation spectroscopy (FLCS). The photoactivity of bR was confirmed through chronoamperometry, affirming that bR retains its functionality forming a micro-photoactive device with stable and notably high and reproducible photocurrent switching over a wide range of flicker frequencies

## Experimental Section

### Materials

1,2-Dioleoyl-*sn*-glycero-3-phosphocholine (DOPC) [purity (>99%)] was purchased from Avanti Polar Lipids (Alabama, USA) and used without further purification. 1,2-Dioleoyl-*sn*-glycero-3-phosphoethanolamine-labeled ATTO655 (DOPE-ATTO655) and NHS-ester-ATTO532 were purchased from ATTO-TEC GmbH (Siegen, Germany). bR (lyophilized purple membrane) was purchased from Bras del Port S.A. (Alicante, Spain). bR structure and purity were manufacturer-guaranteed, as presented in Figures S1 and S2. Phosphate buffer saline (PBS) tablets and Triton X-100 were purchased from Sigma-Aldrich (Wicklow, Ireland). Biobeads SM-2 were purchased from Bio-Rad laboratories (Hercules, CA, USA). Aqueous solutions were prepared using Milli-Q water (Millipore Corp., Bedford, USA). The polydimethylsiloxane (PDMS) silicon elastomer was purchased from Dow Corning GmbH (Wiesbaden, Germany) and mixed following supplier instructions. Silicon wafers coated with a 100 nm layer of gold on a 50 Å layer of titanium were purchased from Platypus Technologies (New Orleans, LA, USA). Monodisperse polystyrene (PS) latex sphere with a diameter of 1.00 ± 0.03 and 4.61 ± 0.4 μm was obtained from Bangs Laboratories Inc. (Fishers, IN, USA). The commercial cyanide-free gold plating solution (TG-25 RTU) was obtained from Technic Inc. (Cranston, RI, USA).

### Preparation and Fluorescent Labeling of bR

bR from the purple membrane was solubilized in PBS buffer (pH 7.4) in the presence of Triton X-100 (5 mM) and kept under gentle shaking for 24 h at room temperature in the dark. The solution was then centrifuged for 1 h at 10,000 rpm to ensure that insoluble particles or impurities were removed. The supernatant was collected and stored at 4 °C and used within 30 days. For FLCS studies, bR was labeled with ATTO-532 by NHS-ester coupling following the protocol provided by ATTO-TECH. Briefly, 1 mL of bR (1 mg/mL) in PBS buffer (pH 8.3) was reacted with NHS-ester-ATTO-532 (1 mg/mL, DMSO) at a molar ratio of protein-to-dye of 1:3. The protein/dye mixture was gently agitated for 1 h in the dark at 20 °C. Unreacted dye was dialyzed from the labeled protein solution with a size exclusion membrane (10 kDa) (Milipore, Ultracel 10) by centrifugation at 10,000 rpm for 30 min. This process was repeated five times, and the labelling efficiency of the final bR-ATTO532 was verified by UV–vis (see Figure S1).

### Reconstitution of bR into Large Unilamellar Vesicles

Reconstitution of bR into liposomes was accomplished using a protocol previously reported by Rigaud et al.^28^ Briefly, bR was inserted into detergent-destabilized pre-formed DOPC liposomes. Detergent was subsequently removed by adsorption on PS beads (biobeads) (see Scheme S1). First, a DOPC lipid film (4 mg) was dried in an amber glass under a gentle nitrogen flow and further dried under vacuum for 1 h. For fluorescence studies, DOPE-ATTO655 was added to the lipid film at a concentration of 0.01 (mol %) before drying the lipids under nitrogen flow. The lipid film was suspended by vortexing the lipid film in 1 mL of PBS buffer (pH 7.4) to achieve a liposomal concentration of 4 mg/mL for about 60 s. The obtained liposomal solution was extruded 11 times through 100 nm polycarbonate membrane (Avanti Polar Lipids). The resulting large unilamellar vesicles (LUVs) were then destabilized with Triton-X100.^29^ The optimal detergent concentration was determined using dynamic light scattering (DLS) and UV–vis (see Figure S2) to be 4.5 mM Triton X100 which was combined with the DOPC liposomes under gentle stirring for 10 min. Then, the appropriate quantities of bR were added to the stirred solution to achieve the desired L/P ratio, and the mixture was kept under gentle agitation for 1 h. The detergent was removed from the proteoliposomes by the sequential addition of four aliquots of pre-washed PS beads (80 mg/mL) every 1.5 h. The proteoliposomes containing labeled bR-ATTO532 were characterized by DLS, FLCCS, and UV–vis spectroscopy, and relevant data were compared to the properties of the liposomes prior to bR reconstitution (see Scheme S1).

### Fabrication of PDMS and Gold Microcavity Arrays

**Figure 1:**
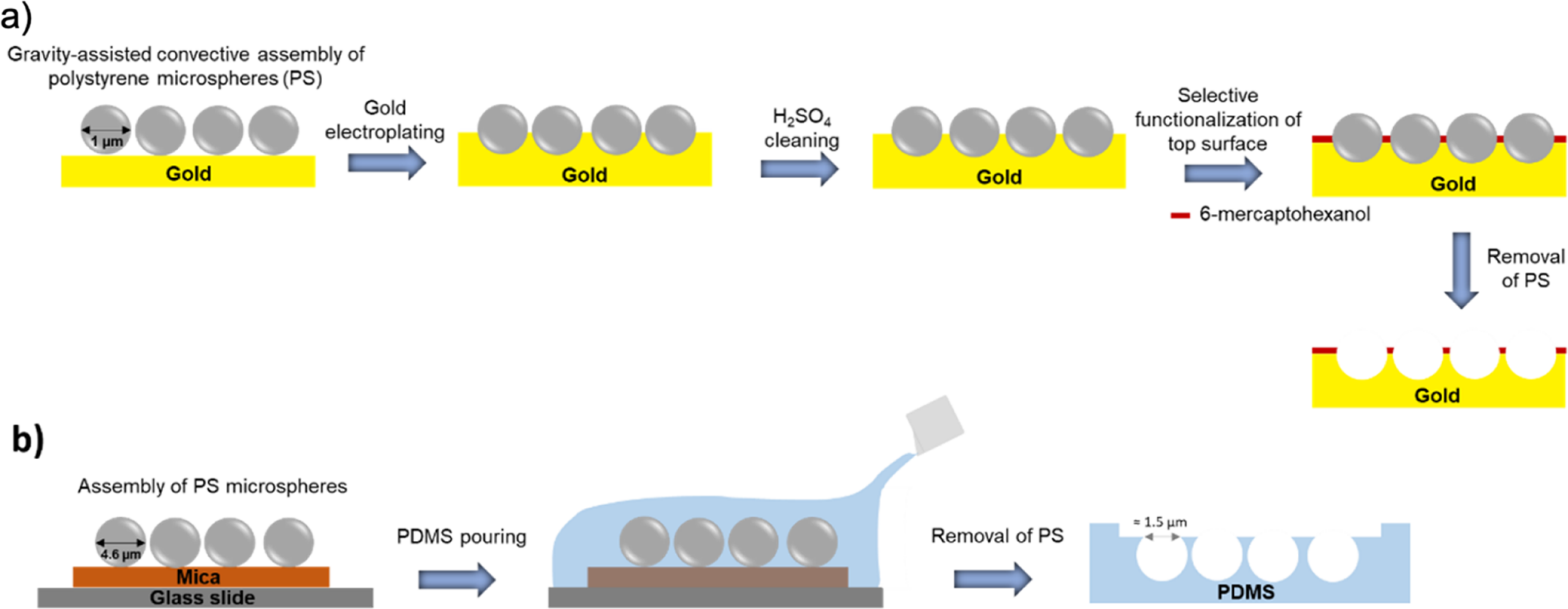
Schematic representation of step-by-step fabrication of gold and PDMS microcavity arrays. (a) To obtain a hemisphere microcavity array, gold was electroplated until the equator of PS, and gold microcavity arrays were prepared using the gravity-assisted convective assembly of PS microspheres followed by gold electroplating. The substrates were functionalized with SAM of 6-mercaptohexanol. (b) Microcavity arrays used in FLCS were prepared by pouring PDMS to pre-deposit PS spheres followed by PS removal and oxygen plasma treatment.

MSLBs were prepared across periodic pore arrays prepared in PDMS for the fluorescence correlation study or in gold for electrochemical experiments by PS sphere templating methods previously described.^30−32^ Briefly, the gold microcavity arrays were prepared by drop casting PS microspheres of 1 μm of diameter followed by gold electroplating, as described in the schematic presented in Figure 1a.^33−35^ To obtain a highly packed microcavity array, highly closed packed monolayers of PS microspheres were cast using the gravity-assisted method onto pre-cut rectangles of gold-coated silicon wafers. Then, gold was electrodeposited to the interstitial surface between the PS microspheres by applying a reduction potential (−0.6 V, Ag/AgCl) to the gold array in the presence of a cyanide-free gold solution. The electrodeposition was controlled by the evolution of the current at the gold array until the current reached a minimum value corresponding to the closer distance between the spheres, indicating that the electrodeposition of gold has reached the hemisphere of PS (Figure S3a).^30^ After the gold electrodeposition, the arrays were electrochemically cleaned using cyclic voltammetry in sulfuric acid (10 mM) for six cycles (−0.2 to 1.8 V) and rinsed with deionized water and ethanol and dried gently under nitrogen flow (Figure S3b). The top surface of the gold microcavity arrays was then selectively functionalized with a SAM of 6-mercaptohexanol (1 mM) for at least 24 h in ethanol (Figure S3c), before removal of the templating spheres which were subsequently washed out of the array with tetrahydrofuran (THF).^30^

The PDMS microcavity arrays were prepared by drop casting 50 μL of ethanol containing 0.1% of 4.61 μm PS spheres (Bangs Laboratories) onto a 1 cm × 1 cm hand-cleaved mica sheet (Figure 1b). After ethanol evaporation, PDMS was poured onto the PS sphere array and cured at 90 °C for 1 h. The microcavity array is then formed after removing the inserted PS spheres by sonicating the PDMS substrate in THF for 15 min. The substrates were then left to dry overnight. Prior to lipid bilayer formation, the substrates were plasma cleaned using oxygen plasma for 5 min, and microcavities were buffer filled before lipid monolayer deposition by sonicating the PDMS substrate in PBS buffer (pH 7.4) for 1 h. As previously reported, this step is important to increase the hydrophilicity of the substrate.^27^

### Characterization of Au/PDMS Microcavity Arrays

The shape and size of the formed microcavity arrays were characterized by field emission scanning electron microscopy (FESEM) and scanning electron microscopy (SEM). FESEM images of gold arrays (top view, tilted, and profile) were obtained using a Hitachi S5500 (Figure S4). All images were acquired using the secondary electron mode. SEM images of PDMS arrays were collected using a Hitachi S3400n, tungsten system using a 5.00 kV accelerating voltage (Figure S5).

### Preparation of Microcavity-Supported Lipid Bilayers Containing Bacteriorhodopsin (bR-MSLBs)

bR-MSLBs were spanned across buffer-filled microcavity arrays using a combination of Langmuir–Blodgett (LB) and vesicle fusion methods, as described previously.^32,33^ Briefly, approximately 50 μL of DOPC (1 mg/mL in chloroform) was deposited onto the air–water interface of LB trough (NIMA 102D) and the solvent allowed to evaporate for 15 min. The resulting lipid monolayer at the air–water interface was compressed four times to a surface pressure of 36 mN/m at 15 mm/min. Then, the microcavity arrays were immersed into the LB trough until all of the cavities were submerged completely into the subphase. The microcavity array was then withdrawn from the trough at a rate of 5 mm/s, while the surface pressure of the lipids was retained at 32 mN/m to ensure an adequate transfer of the DOPC monolayer. To assemble the upper leaflet of the bilayer and reconstitute bR into the MSLB, the lipid monolayer was exposed to the aforementioned DOPC/bR proteoliposomes (0.25 mg/ml) and allowed to incubate for 3 h in the dark. The integrity of spanning lipid bilayers was established by FLCS, and the photoelectrical response of bR-MSLBs was studied by chronoamperometry.

### FLCCS and FLCS Measurements

Single-point FLCCS measurements were performed on the prepared proteoliposomes containing labeled bR-ATTO532 and DOPE-ATTO655 to confirm the incorporation of bR into liposomes and the formation of proteoliposomes. FLCS was also employed to evaluate the lipid bilayer formation over micropores after proteoliposome fusion by measuring the diffusivity of DOPE-ATTO655. bR integrity and proper insertion in MSLBs were confirmed by monitoring the diffusivity of bR-ATTO532. Fluorescence measurements were performed on a MicroTime 200 lifetime (PicoQuant GmbH, Berlin, Germany) using a water immersion objective (NA 1.2 UPlanSApo 60 × 1.2 CC1.48, Olympus). The detection unit comprises two single photon avalanche diodes from PicoQuant. A labeled lipid membrane marker DOPE-ATTO655 was excited with 640 nm LDH-P-C-640B (PicoQuant), and bR-ATTO532 was excited with a 532 nm PicoTA laser from Toptica (PicoQuant). To exclude scattered or reflected laser light, emitted fluorescence was collected through an HG670lp AHF/Chroma or HQ550lp AHF/Chroma band pass filter for 640 or 532 nm lasers, respectively. A 50 μm pinhole was used to eliminate photons from outside the confocal volume. Before FCLS measurements, backscattered images of the substrate were taken using an OD3 density filter to ensure the optimal positioning of the focus to the center of the microcavity. Then, the bilayer position was determined by *z*-scanning until the point of maximal fluorescence intensity of DOPE-ATTO655 was found. At this point, the fluctuating fluorescence intensity of the labeled lipid marker or bR-ATTO532 was measured for 30 to 60 s per cavity, and replicate measurements were collected from 20 to 30 cavities per sample. To assess the diffusion time (ms) and the fluorescence lifetime (ns), the emitted photons were analyzed by a time-correlated single photon counting system (PicoHarp 300 from PicoQuant). The fluorescence fluctuations obtained are then correlated with a normalized autocorrelation function (eq 1)
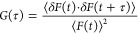
1

The autocorrelation curves obtained from the fluorescence fluctuations of DOPE-ATTO655 and bR-ATTO532 were fitted to a 2-D model (eq 2) using the software SymphoTime (SPT64) version 2.4 (PicoQuant).
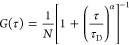
2Here, ρ represents the amplitude at *G*(τ) and is defined as the inverse of number of molecules (1/*N*), α is the anomalous parameter, and τ_D_ is the diffusion time of the fluorescent marked molecules in the lipid membrane. The diffusion coefficient is related to the correlation time τ_D_ by the relation *D* = ω2/4τ*D*, where ω is the 1/*e*^2^ radius of the confocal volume, that is, the waist of the exciting laser beam. ω was measured for each excitation wavelength using a reference solution of free dye for which the diffusion coefficient is known. The ω was determined by calibration using reference dyes; ATTO-655 (Atto TEC, GmbH) for a 640 nm laser and rhodamine 6G for a 532 nm laser at 20 °C in water.

### Photocurrent Generated by bR-MSLBs

Supported lipid bilayers over gold microcavity arrays were investigated using electrochemical impedance spectroscopy (EIS) performed on a CHI 760B bipotentiostat (CH Instruments Inc., Austin, TX) in a three-electrode cell consisting of Ag/AgCl (1 M KCl) as the reference electrode (RE), a platinum coiled wire as the counter electrode (CE), and the gold-MSLB as the working electrode (WE). Impedance measurements were performed in 0.1 M KCl as supporting electrolyte solution.

The EIS data were recorded over the frequency region 10^4^ to 10^–2^ Hz at 0 V versus Ag/AgCl. The impedance spectra were fit to an equivalent circuit, as previously described.^33,34,36^ In the circuit, *R*_S_ is the solution resistance, *R*_M_ and CPE_M_ represent the resistance and constant phase element (CPE) of the membrane, and *R*_C_ and CPE_C_ represent the cavity resistance and CPE of the gold substrate. CPEs were used in the equivalent circuit instead of pure capacitors to account for the heterogeneity of the WEs which is expected to deviate from pure capacitor behavior due to their porous arrays and the assembled bilayer membrane (Table S1 and Figure S6a).

**Figure 2:**
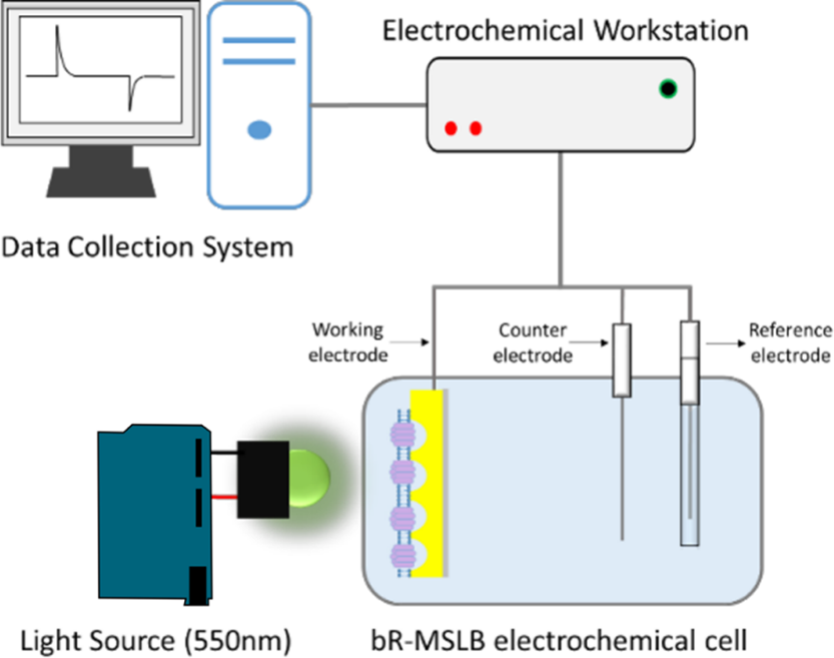
Schematic representation of the experimental setup used for photocurrent measurements of MSLBs containing bR (bR-MSLBs). The electrochemical apparatus consisted of Ag/AgCl as the RE and a platinum wire as the CE. The WE comprises the MSLB-bR.

The photocurrent was generated by photoactivating bR-MSLBs with a 2 mW light-emitting diode (LED) (λ = 555 nm) (Thor Labs, England) kept inside the Faraday cage, and the LED was operated using a microcontroller “Arduino Uno” (Arduino, Italy). The source of light was maintained 1 cm from the substrate. The experimental setup is shown in Figure 2. The photocurrent data were monitored at potential DC bias of 0 V (vs Ag/AgCl 1 M KCl). All measurements were conducted in 0.1 M KCl as the supporting electrolyte.

## Results and Discussion

### Preparation and Characterization of Fluid bR-MSLBs

The MSLBs were prepared and characterized, as reported in detail previously.^32,33,37^ Previous data confirm that surface-modified pore arrays in gold and PDMS, that are pre-aqueous filled with buffer, support stable, hydrated lipid bilayers, where the bilayers are fluidic and span the cavity apertures. Also, both lipid and any reconstituted protein exhibit liposome-analogous diffusion coefficients.^32,33,37^ It was also confirmed previously that the method of bilayer preparation results in a single bilayer^38^ and that the bilayer has sustained integrity, denying access to non-permeable species into the cavity over the experimental window used here.^39^ Here, the LB monolayer and liposome fusion method reported previously was used to create the MSLBs, but in this instance, the liposomes contained reconstituted bR.^27,33^ The bR proteoliposomes used for fusion were characterized by DLS (see the Supporting Information), and the protein reconstitution into liposomes was confirmed in solution (PBS, pH 7.4) before the liposomes were used for bilayer formation, using FLCCS.

**Figure 3:**
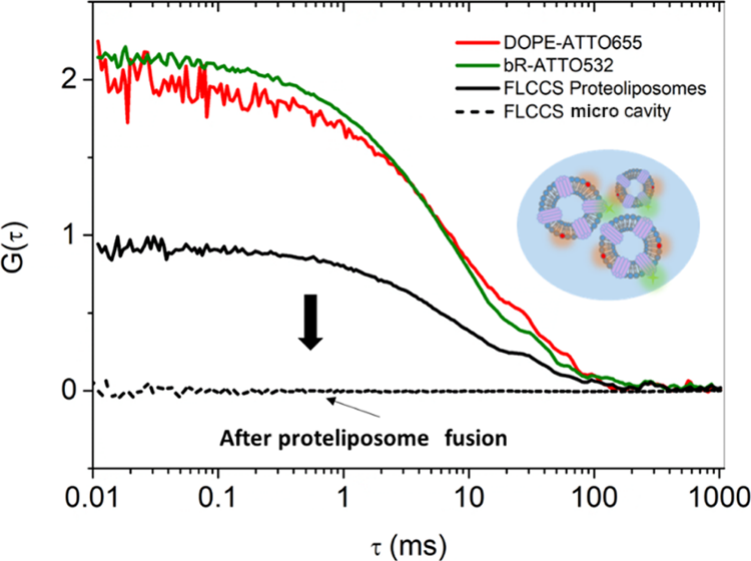
Autocorrelation functions from bR-reconstituted DOPC proteoliposomes monitoring the DOPE-ATTO655 (0.01 mol %) (red) and bR-ATTO532 (0.01 mol %) labels (green) before disruption at the microcavity lipid monolayer interface. Black lines show the cross-correlation curves before (solid line) and after (dashed line) proteoliposome fusion to the DOPC monolayer, that is, following lipid bilayer formation. The insert shows the proteoliposomes containing labeled bR-ATTO532 (green tagged purple protein) and DOPE-ATTO655 (red tagged lipid), illustrating how the cross-correlation signal is only observed if the bR is reconstituted into the liposomes.

To confirm that the bR was reconstituted into the liposomes, the proteoliposomes were labeled with a lipid marker, and the signal from this was cross-correlated with the FCS signal from the bR label to ensure that they co-diffuse, that is, to confirm that they are both reconstituted into the same liposome. Using FLCCS, the diffusivity of the green labeled bR and red labeled DOPE though the confocal volume was evaluated simultaneously.^40^Figure 3 shows the FLCCS of proteoliposomes tagged with DOPE-ATTO655 and bR-ATTO532 (Figure 3, schematic). The amplitude of the cross-correlation signal *G*(τ) (Figure 3, black line) is characteristic of concomitant movement of protein and tagged liposomes and indicates that both labeled DOPE and bR are diffusing together within the laser confocal spot, consistent with protein reconstitution into proteoliposomes.^40,41^ After proteoliposome fusion to the DOPC monolayer to form the lipid bilayer, signal cross-correlation *G*(τ) is lost, as expected, indicating lipid and protein mixing into the MSLB and rupture of the proteoliposome.

**Figure 4:**
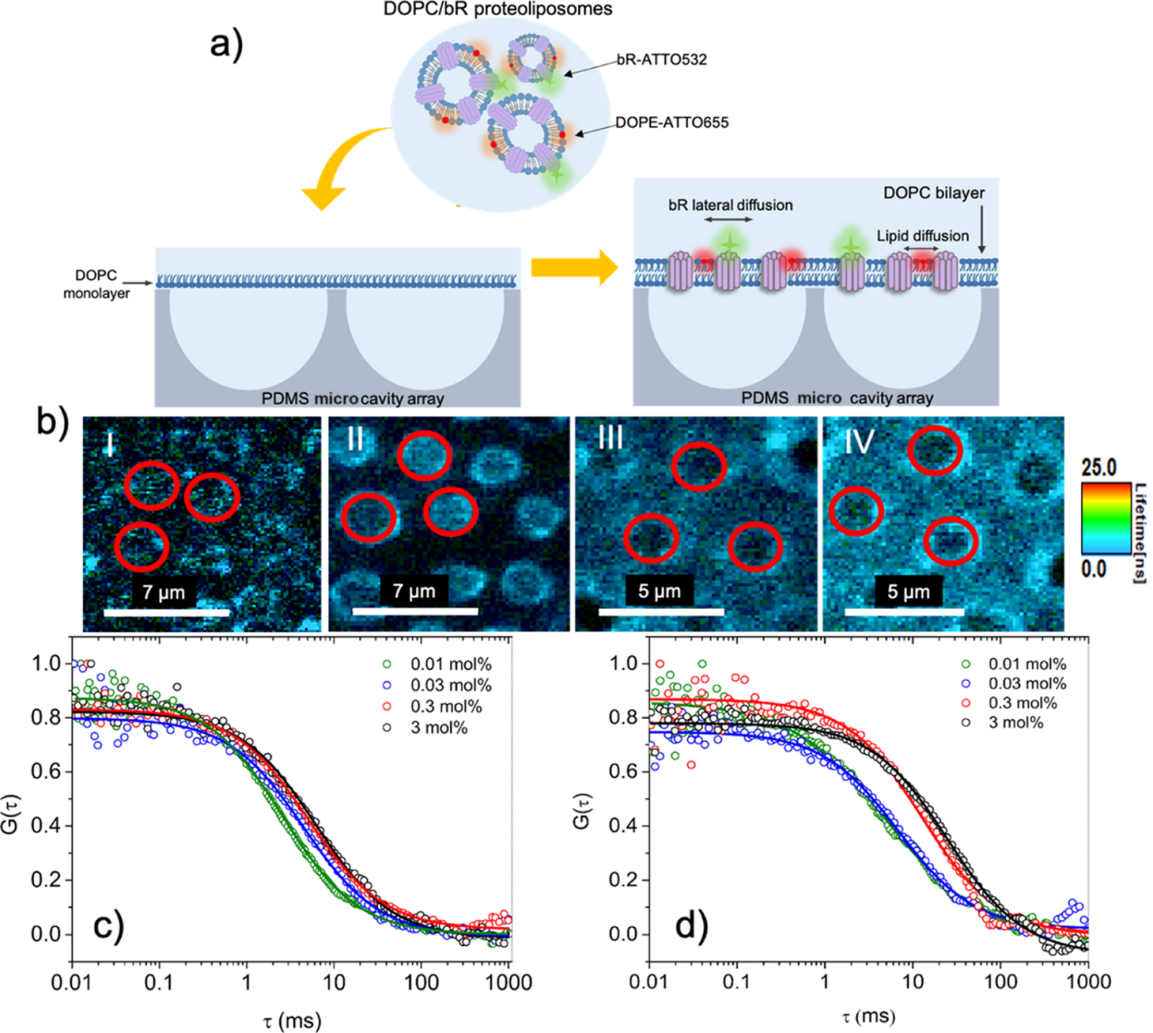
(a) Schematic representation of preparation of MSLBs containing bR spanned over aqueous buffer-filled PDMS microcavity arrays using LB/proteoliposome fusion. (b) (I–IV) FLIM of DOPE-ATTO655 obtained for bR-MSLBs obtained after proteoliposome fusion containing different concentrations of bR, for 0.01, 0.03, 0.3, and 3 mol %, respectively. The red circles indicate the rim of microcavities from within which the FLCS signals were collected. (c) and (d) FLCS obtained using labeled DOPE-ATTO655 and bR-ATTO532.

Membrane proteins provide outstanding opportunities for purposing sophisticated molecular switches. However, their membrane environment is key to their function. In this context, the fluidity of MSLBs is a key advantage of MSLBs over solid-supported lipid membranes. Therefore, to investigate the photoactivity of bR in the MSLBs, the lipid bilayers were spanned across microcavity arrays using a hybrid method combining LB lipid monolayer deposition with proteoliposome fusion reported previously.^27^ Proteoliposomes comprising DOPC and reconstituted with different concentrations of bR were disrupted at the aqueous-filled microcavity arrays modified with an LB-transferred DOPC monolayer (Figure 4a). The formation of the MSLB was evaluated by fluorescence lifetime imaging (FLIM) and by monitoring the lateral diffusion of labeled DOPE-ATTO655 using FLCS. Imaging and diffusion values conformed to previous reports for MSLBs with FLIM images of DOPE-ATTO655 obtained for DOPC/bR-reconstituted lipid membranes, confirming that a continuous lipid bilayer spans the PDMS microcavity array and that the bilayer was faithfully formed with controlled concentrations of bR reconstituted. The rim of the microcavity is marked in the figure by the red circle (Figure 4bI–IV), and the fluorescence from the labeled protein evident above the microcavity pores indicates that the bR is reconstituted into the membrane spanned over the micropores.

Using FLCS, a highly sensitive single molecule technique, the lateral diffusion coefficient of DOPE-ATTO655 obtained at DOPC/bR bilayers shows that the reconstitution of bR into the DOPC membrane affects the diffusivity of DOPE. This effect scales with the protein concentration, where DOPE lateral diffusion decreases as the protein content increases in the bilayer (Table 1). The lateral diffusion coefficient obtained for a DOPC lipid bilayer without bR (bare DOPC) was measured as approximately 10 μm^2^·s^–1^. This value is consistent with previously reported by our group for DOPC MSLBs as well as for other reported free-standing DOPC bilayers and liposomes.^27,33,42^ The lateral diffusion of labeled DOPE was approximately 9.8 ± 0.5, 8.4 ± 0.6, 6.5 ± 0.4, and 5.5 ± 0.6 μm^2^·s^–1^, for lipid bilayers containing 0.001, 0.03, 0.3, and 3 mol % of bR, respectively. These values confirm lipid bilayer formation and indicate the insertion of the protein to the lipid membranes. The progressive decrease in lipid diffusion with the increasing bR content is consistent with behavior noted for bR at liposome models and attributed to the impact of the increasing protein/lipid ratio on membrane viscosity.^43^ For the MSLB reconstituted bR-ATTO532 (Figure 4c), a diffusion coefficient of 4.2 ± 0.3 μm^2^·s^–1^ for the lowest bR concentration (0.01 mol %) was obtained. The reduced diffusion coefficient of bR compared to the lipid is consistent with the large radius of this protein, which, as described, is a seven-pass transmembrane protein spanning both lipid leaflets of the membrane. Translational diffusion of proteins in biological membranes has been described by the Saffman–Delbrűck (SD) relation, a hydrodynamic model that treats the bilayer as a 2-D, viscous continuum interfaced with an infinite volume of fluid through which a solid cylindrical shape (representing the protein) diffuses (eq 3).^44,45^ The model suffers a number of limitations both when dimensions and density of protein exceed certain limits, and in models that show reduced fluidity like SLBs, but was used here as our platform offers close to a continuous planar membrane decoupled from the surface.^46^ Using the SD model, we estimated the hydrodynamic radius of bR within the DOPC MLSB of 4 nm for 0.01 mol % and 4.7 nm for 0.03 mol %,^45^ and our measured diffusion coefficient agrees well with those determined in freestanding membranes of pore-spanning membranes, giant unillamelar vesicles (GUVs) and black lipid membranes (BLMs).^47−49^
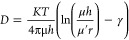
3

**Table 1:** Diffusion Co-efficient of Labeled bR-ATTO532 (*D*_bR_) and DOPE-ATTO655 (*D*_DOPE_) Introduced in the Lipid Bilayer Comprising DOPC Using LB Lipid Transfer Followed by Proteoliposome Fusion^a^

bR concentration (mol %)	*D*_bR_ (μm^2^/s)	*D*_DOPE_ (μm^2^/s)
0.01	4.2 ± 0.3	9.8 ± 0.5
0.03	3.4 ± 0.6	8.4 ± 0.6
0.3	1.2 ± 0.4	6.5 ± 0.4
3	1.0 ± 0.5	5.5 ± 0.6

a The concentration of bR varied during proteoliposome preparation. The α co-efficient was determined as approximately 1.0 for all measurements.

The diffusion coefficient of bR was observed to decrease with the increasing bR concentration in the membrane (Figure 4d). This correlated with reduced labeled DOPE diffusion with increasing protein concentration described above (Table 1). It is notable that the anomalous co-efficient, α, remained approximately 1 across all protein concentration, indicating, that the effect is from viscosity changes rather than, for example, protein aggregation. In conclusion, our results clearly demonstrate that bR reconstitutes properly into the MSLB and that the membrane components of MSLBs show high lateral mobility at the freestanding microspore array, an important requisite to guarantee protein mobility and function.

### Photoactivation of bR Incorporated into DOPC Lipid Bilayers and Time Resolution of Light-Induced Current

**Figure 5:**
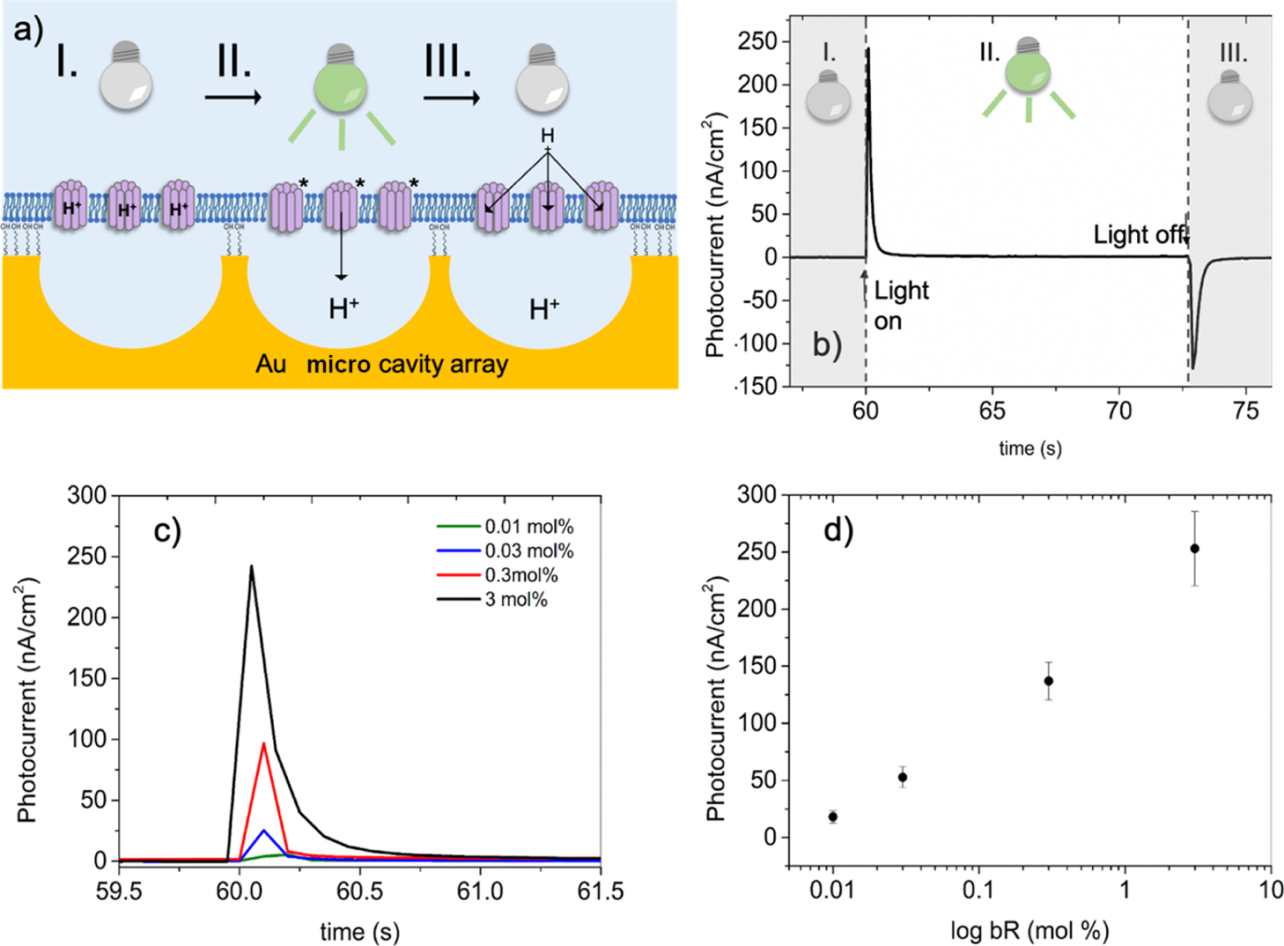
Representative photocurrents recorded from bR-MSLBs. (a) Schematic representation of photocycle mechanism of bR at MSLBs: (I) represents the ground state of bR without presence of light, (II) indicates the photoswitch of bR in the presence of light, represented by its excited state and the subsequent proton release, and (III) return of bR to the ground state when light is switched off. (b) Typical photocurrent decay of bR-MSLBs comprising DOPC/bR (3 mol %) (KCl 0.1 M, pH 5.6) (I) before photoactivation, (II) photoelectrical response of proton release, and (III) proton uptake. (c) Photoelectrical response of proton release generated with different bR concentrations (KCl 0.1 M, pH 5.6). (d) Photocurrent of bR-MSLBs indicating a logarithm dependence of *i* vs bR concentration.

To determine that protein functionality is intact and to characterize the light-induced proton flow, bR-reconstituted bilayers spanned over gold microcavity arrays were evaluated under dynamic light activation by chronoamperometry. EIS measurements were performed at 0.0 V bias in the dark, and data indicate that in comparison to pristine bilayers, the presence of bR alters the double-layer properties of the membrane, reducing membrane resistivity while increasing the capacitance of the membrane (Figure S5). For chronoamperometric measurements, the three-electrode system, as illustrated in Figure 2, was placed in a closed Faraday cage, and the electrochemical cell was allowed to equilibrate for 300 s in the dark before photoactivation of bR. Then, the current in the electrochemical cell was measured for 60 s to obtain a dark current of approximately 0.1 nA/cm^2^ prior to irradiating the sample with an LED light (2 mW, λ_em_ 555 nm) for approximately 10 s. The dark current was subtracted from the generated photocurrent measured for the bR-MSLBs. The photocurrent was related to the area of substrate covered by the lipid bilayer, which was approximately 1 cm^2^. Therefore, the current per cm^2^ was calculated, as previously reported for porous substates.^19^Figure 5b shows a characteristic photocurrent response from the bR-MSLBs. When the light is switched ON, an anodic photocurrent evolves to a peak current maximum of approximately 240 nA/cm^2^ that then decays to a steady-state current of 0.1 nA/cm^2^. Under illumination, bR retinal isomerizes with a high quantum yield, from the all-trans conformer to the 13-cis isomer initiating the proton transport process, the entire cycle takes roughly 15 ms, leading to proton transfer from the distal to proximal side of the bilayer. Given the cycle time, bR is expected to undergo multiple cycles of photoexcitation during the illumination which lasts approximately 10 s. Therefore, the regeneration of bR reaches saturation where proton release and uptake reaches an equilibrium. When the light is switched OFF, the concentration of excited state rapidly diminishes and proton reuptake takes place.^50^

Under continuous illumination, the decay of the peak current can be attributed to accumulation of the M intermediate and saturation of proton transport. On switching off the light, a cathodic current evolves with a maximum of density of −125 nA/cm^2^ which also decays to baseline. The decay data were analyzed by fitting each peak current (ON/OFF) to a biexponential function
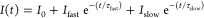
4where *I*_0_ represents the base line of the photocurrent at steady state, *I*_fast_/*I*_slow_ indicates the amplitude of the intermediate decay, *t* is the duration of the decay, and τ is the decay constant of the current signal. The data for the kinetic decay of the photocurrent peaks obtained after fitting to eq 4 are displayed in Table S2, and the fitted decays are shown in Figure S6. For the photoactivation process, the fast component of the decay is below the limit of the resolution of the measurement, but both components of the decay, within experimental error, showed no dependence on the protein concentration, and the photocurrent amplitude of *I*_fast_ corresponds to approximately 85–90% of the photocurrent signal. This independence is anticipated as the decay time is expected to depend on the incident light intensity and is a convolution of kinetic response from the proton pump, and the electrical circuit and therefore, is specific to the system.

To confirm that the current signal is coming from the bR proton pump, control experiments were carried out under analogous conditions and confirm that, by comparison, negligible photovoltaic current is observed on irradiation of the bilayer/electrode in the absence of the protein (Figure S6b). Contributions from conductive artifacts were excluded by evaluating the impact of changing the electrochemical photoactivation apparatus, such as by changing the position of the light source while monitoring the current, we did not observe any other contributions to the photoelectrical signal.

The photoelectric signal observed after photoactivation of bR-MSLBs is attributed to proton displacement from the bulk solution across the lipid bilayer toward the interior of the microcavities (Figure 5a) and is similar to the bR photocurrent previously reported in supported structures.^19,51,52^ The stationary light-induced electric current observed (Figure 5b) is likely capacitive current from the underlying gold array and indicates non-random orientation of bR at the membrane.^53^ Crucially, the observation of the characteristic transient photocurrent confirms that bR retains its functionality when integrated into the MSBLs, and the observation of two transient peaks suggests that in each case, vectorial proton transport occurs. The observed photocurrent and low leakage current (0.1 nA cm^–1^) also indicate that the MSLB membrane is intact and proton impermeable.^19,24^ This is confirmed further below, in experiments where pH of the contacting solution at the distal leaflet is changed.

It is notable that in the dynamic photocurrent response, as shown in Figure 5c, the ratio of the peak magnitude of the light on versus light off currents is approximately 1.8. Notably, the photocurrents, normalized to electrode area observed are comparable or indeed significantly higher than related reported systems, particularly given the light intensity in our experiments is low, compared to other reports. For instance, under optimal conditions and using high power systems (250 W), Horn and Steinem obtained a photocurrent of 250 nA/cm^2^, Guo et al. obtained a photocurrent of 350 nA/cm^2^, and Lu et al. obtained a photocurrent of 120 nA/cm^2^.^19,54,55^ This, we tentatively attribute to the fact that in the present system, the bR is reconstituted into a single bilayer rather than a layer-by-layer assembly, therefore bR light activation directly pumps the proton across the membrane between bulk and cavity solution, with no intervening structures such as additional bilayer or multilayer impeding the proton transport.

The magnitude of the photocurrent is influenced by the concentration of bR in the proteoliposomes used to prepare the MSLB. As shown by the overlay of the photocurrent response from four MSLBs prepared from liposomes with different protein concentrations (Table 2, Figure 5c), a logarithmic correlation is observed with the concentration of protein (log [bR]) (Figure 5d). The quantitative relationship between current and the bR concentration in the fusion proteoliposomes result speaks to the robustness of the reconstitution method, indicating complete transfer of reconstituted protein to the bilayer and retention of is electro/photoactivity within the bilayer. As noted, for DOPC only (bare MSLB), the photovoltaic response contributes less than 2% to the photocurrent generated by bR (Figure S6b).

**Table 2:** Photocurrent Activity of bR Incorporated into Gold-MSLBs Activated with 2 mW LED (555 nm) in KCl (0.1 M, pH 5.5)

bR concentration (mol %)	*i*_release_ (nA/cm^2^)	*i*_uptake_ (nA/cm^2^)
0.01	18.6 ± 5.4	–10.1 ± 3.7
0.03	52.7 ± 9.2	–32.1 ± 1.6
0.3	137.4 ± 16.4	–86.9 ± 6.5
3	253.5 ± 32.3	–129.5 ± 16.8

### Photoelectronic Response of bR-MSLBs to Asymmetric pH Gradient

The bR proton pump cycle is initiated by photoisomerization of all-*trans* to 13-*cis* retinol caged at the center of the bR within the membrane.^56,57^ This photoinduced conformational change initiates a photocycle that through a series of spectroscopically distinguishable intermediates leads to proton release at the extracellular side of the membrane between the L and M steps of the cycle, which is then followed by the reprotonation of the Schiff base that is preceded by a step whereby water is inserted temporarily into the proton channel and protonates through a proton chain mechanism.^58,59^ This process requires significant conformational/structural changes to the channel helices. The pH of the contacting solution can not only alter the proton concentration in the bulk solution but also influences the proton release of bR. For instance, several studies have shown that the rate of deprotonation of bR decreases with decreasing pH.^60,61^

**Figure 6:**
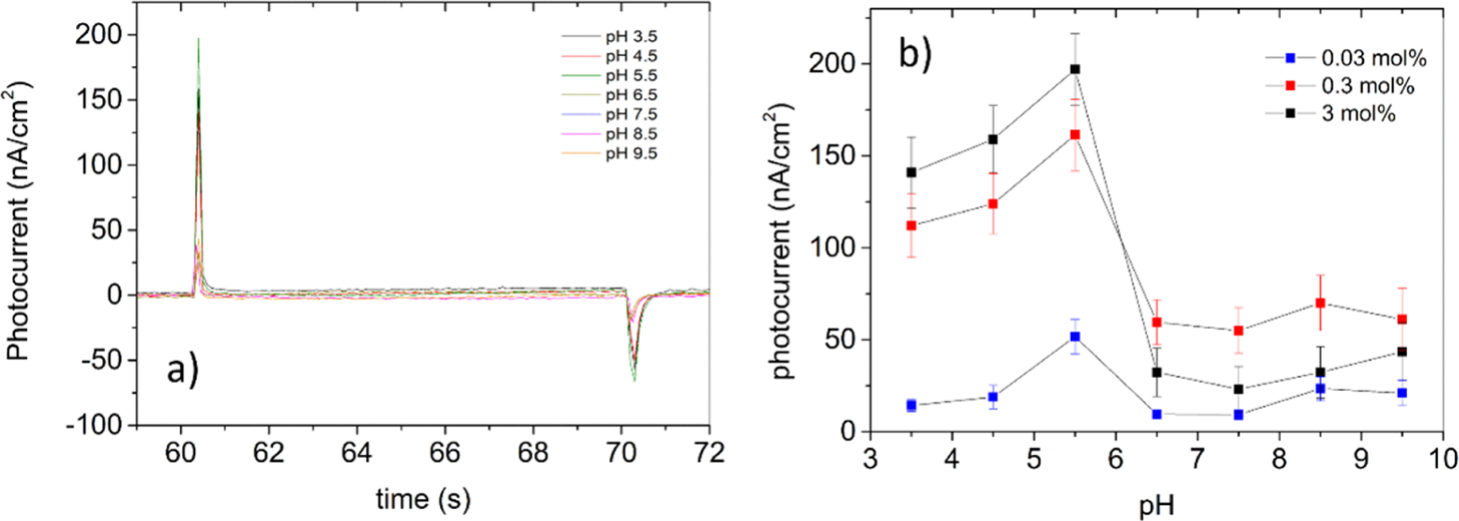
Photocurrent obtained after activation of bR at different bulk pH values. (a) Photocurrent obtained for bR-MSLBs comprising DOPC and doped with bR (3 mol %). (b) Photoactivity of bR-MSLBs at different bR concentrations. The solid lines are guides for the eye. These measurements were taken at 20 ± 2 °C.

To investigate the proton switching of bR at MSLBs as a function of pH, the photoelectrical response of bR was analyzed, where the pH of contacting electrolyte solution in the electrochemical cell was modified by addition of HCl (1 mM) or NaOH (1 mM) over the range of 3.5–9.5 (Figure 6a). Note that in these experiments, the pH of solution in the microcavity at the proximal membrane interface is 7.4, and it is only the pH of the contacting solution at the distal leaflet that is changed. Following pH adjustment, the samples were allowed to equilibrate at each new pH for 300 s in the dark before exposure to light and before the photoactivity of bR was recorded. We and others have reported previously that a pH gradient can be sustained at artificial bilayers^34^ for windows of about an hour, and the samples here were analyzed within 300 s to ensure that the gradient is sustained within the experimental window.^62^ As expected, the photoactivity of bR in DOPC MSLBs is strongly influenced by the pH of the contacting solution. The lowest photocurrent was observed at basic pH (>8.5), and maximum photocurrent was recorded at pH 5.5 which actually decreased at more acidic pH values (<5.5), as shown in Figure 6b. This pattern is similar to previous reports for supported lipid bilayer systems.^63,64^ However, the maximal photocurrent here is observed at an order of magnitude more acidic environment (pH ≈ 5.5) than previously reported (pH ≈ 6.5).^50^ We can relate this effect to the pH gradient that is maintained using the MSLBs,^55^ and it also confirms, consistent with the low dark current described, the excellent insulating properties of MSLBs indicating their suitability for such artificial proton pump devices.^65^

To understand whether pH alters the kinetics of the electrodic response for the photoactivated and dark initiated states, the current decays post light and dark steps were fit to exponential decay curves represented by eq 4 (Figure S7). The results are shown in Table S3. The data fit best to biexponential decays for the photoactivated process and as before, the fast decay, is at the limit of resolution of our measurement and therefore showed no pH dependence. However, the slow step, although only contributing 2% of current amplitude, did show a pH dependence, consistent with photoelectric behavior of bR expressed at cells.^66^

### Photocurrent of bR-MSLB Response of Transient Light Activation

**Figure 7:**
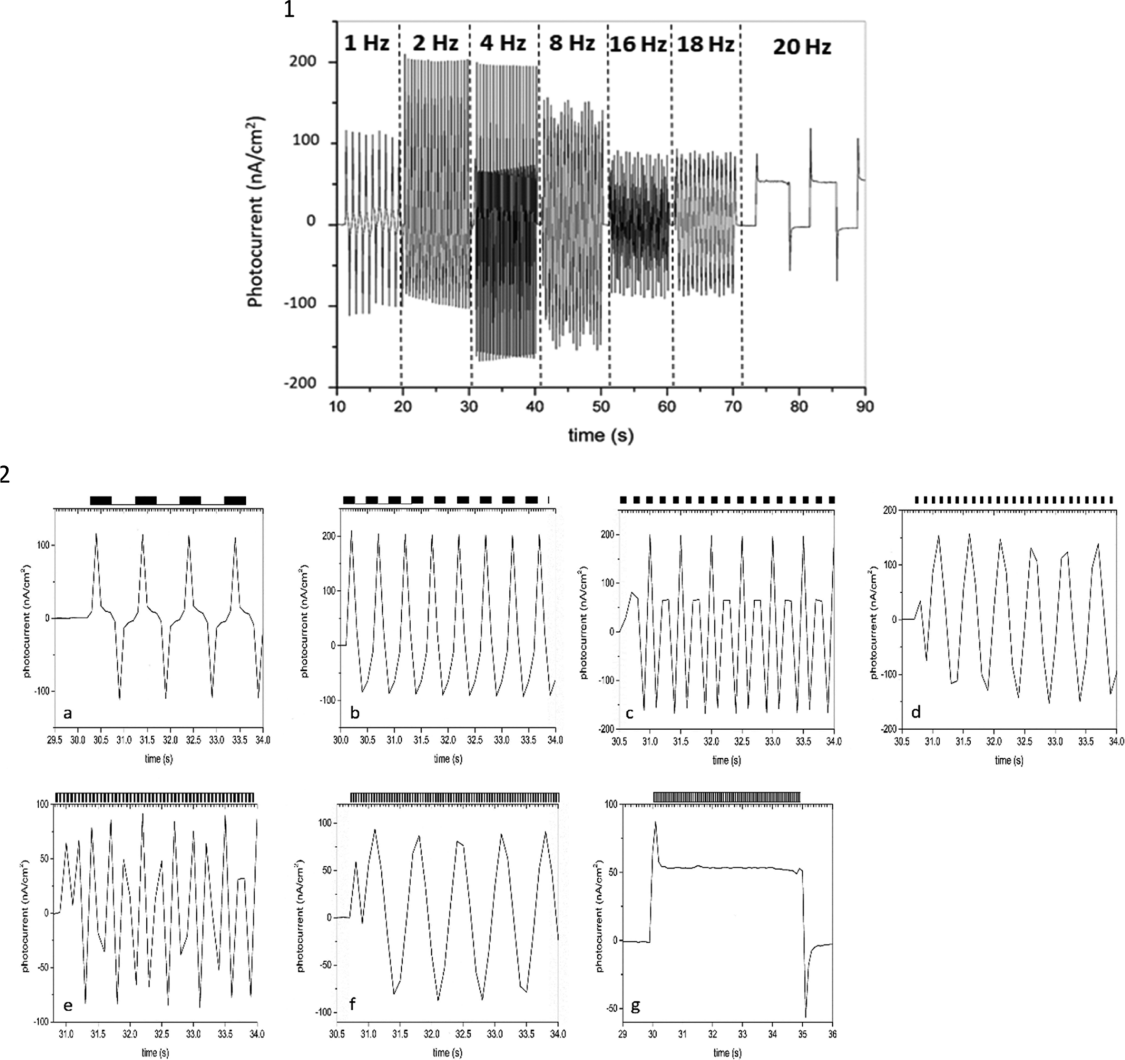
Photocurrent of bR-MSLB generated on the application of a flickering light source. (1) Electrochemical current increases at 2 and 4 Hz but decreases for higher frequencies. At 20 Hz, a stationary photocurrent was observed. (2) Individual *i*–*t* curves for photoactivation at different frequencies (a) 1, (b) 2, (c) 4, (d) 8, (e) 16, (f) 18, and (g) 20 Hz. The bar over the graphs represents the state of activation of light (on, black; off, white).

Photoswitch devices that operate under flickering light conditions are important in the development of artificial biocomponents, such as biotransistors, biocapacitors, and biomimetic artificial retina.^14,55,67,68^ The quasi-stationary photocurrent generated by bR-MSBLs under transient light activation illustrates an important characteristic of the molecular switch in MSLBs. Given the high photocurrent signals under relatively low light intensity we were interested to evaluate the photoactivity of bR-MSLBs under oscillating light activation. Thus, the devices were exposed to a flickering light (1–20 Hz) and the photocurrent was obtained, as displayed in Figure 7. Here, a pulse of light represents the complete cycle of light activation/deactivation (ON/OFF), for instance, 1 Hz (1 pulse per second) represents light ON for 0.5 s, followed by light OFF for 0.5 s, 2 Hz represents 0.25 s of light ON followed by 0.25 s of light OFF, and so on for the other frequencies. The photoactivity of bR incorporated into DOPC MSLB (0.3 mol %) increases for frequencies of 2 and 4 Hz, when compared to 1 Hz. This is possibly due to a synergistic effect, as for 1 Hz, the full photo-circle takes approximately 1 s (Figure 7), it is possible that for 2 Hz, bR excitation is enhanced, indicating that the concentration of excited bR is further increased, with a similar period to the sinusoidal photocurrent of approximately 0.25 ms (Figure 7). For 4 Hz, the first cycle is not completed, forming a hybrid pattern combining a high amplitude of 200 nA/cm^2^ with a low amplitude with approx. 82 nA/cm^2^ (Figure 7). The period of the sinusoidal photocurrent was 0.12 ms. At 8 Hz (Figure 7), the wave period of photocurrent increased by a factor of 2 when compared to 1 Hz, indicating that the lifetime of excited bR was increased; therefore, the steady-state was prolonged. At long time scales, the photocurrent showed a sinusoidal wave pattern with a period of 3.3 s, *A*_max_ of 150 nA/cm^2^, and *A*_min_ of 126 nA/cm^2^ (Figure 7). At 16 Hz (Figure 7), the photocurrent showed multiple amplitudes, with a period of 2.1 s, *A*_max_ of 92 nA/cm^2^, and *A*_min_ of 48 nA/cm^2^. At 18 Hz (Figure 7), the period of the sinusoidal photocurrent was 2.5 s with *A*_max_ of 95 nA/cm^2^ and *A*_min_ of 89 nA/cm^2^. At 20 Hz (Figure 7), the photocurrent showed two distinct phases, an initial peak of approx. 90 nA/cm^2^ followed by a decrease until a steady state. In this case, the steady state current was at 50 nA/cm^2^. This indicates that the proton pump has reached an equilibrium, similar to what was observed in Figure 5b. However, the photocurrent does not decay to base line, indicating that the flux of protons may reach a steady state of 50 nA/cm^2^.

The photocurrent maximizes at 4 Hz, and by 8 Hz, it is observed to decrease as, kinetically, the photocycle of bR is not completed by the arrival of the next photons, similar to previous reports.^63^ However, it is important to note that the device reported by Lu et al. is formed by a thick multilayered bR structure, whereas here, bR is reconstituted into a single biomimetic lipid bilayer. The photocurrent continues to decrease with increasing frequencies until 20 Hz. The current dependence across the range of light activation frequencies shows a pattern observed for a photo-biocapacitor as the current decreases with the light alternation state (ON to OFF). Here, the bR-MSLBs system is demonstrated to convert flickering light impulses below 20 Hz into distinguishable patterns of photocurrent, and a stationary photocurrent was observed at 20 Hz, indicating that bR is continuously active. By controlling the incident light flicker frequency in this way, we have the opportunity to fast switch between amplitude, and controlling the period of the incident light could be used to create a pH gradient across MSLBs.^69^ As previously discussed, the proton gradient observed for the bR containing bilayer is only present momentarily. In order to maintain a proton gradient across the bilayer, fast flicker rates could be instigated.

## Conclusions

A new approach to a switchable photoelectric device is described based on photoactivated light-driven proton transfer across bR reconstituted into a MSLB. Exploiting an MSLB enables reconstitution of native biological switches into a strongly biomimetic and fluidic environment, with interfacial addressability without the need for laborious preparation of mutants and the associated changes that may occur to protein function. A reliable method for reconstituting this seven-pass protein into the MSLB is described exploiting the LB monolayer over pore assembly followed by proteoliposome fusion. FLCS of labeled bR, following proteoliposome fusion, indicates that the protein was successfully incorporated into MSLBs where it was found to diffuse, retaining a high degree of lateral fluidity within the device, with a diffusion coefficient of 4.2 ± 0.3 μm^2^·s^–1^ for reconstituted bR-ATTO532 at 0.01 mol % (in the proteoliposome preparation) at membranes that exhibited a lipid label diffusion coefficient of 9.8 ± 0.5 μm^2^·s^–1^. The diffusion coefficient of both membrane and bR decreased linearly with bR concentrations, attributed to viscosity changes to the membrane indicating faithful reconstitution of different concentrations of bR into the MSLB using the reported reconstitution method.

The photoelectrical properties of bR-MSLBs were evaluated by studying the photocurrent signal generated by bR under temporal and transient light illumination and bR concentration, and pH and light flicker frequency were all found to influence the photocurrent generated. Overall, large photocurrents were observed, considering this is a single bilayer device and the signal scaled with the bR concentration. In addition, the chronoamperometry assays demonstrated a direct relationship between the protein concertation and the current response.

The approach offers a robust and addressable platform that should facilitate further spectroscopic study of the mechanism and dynamics of bR proton transport at a lipid membrane, but more broadly, as bR is a seven-pass protein, its reliable reconstitution into MSLBs suggests comparable proteins of pharmaceutical interest including G-protein-coupled seven-helix receptors may be reconstitued by similar methods and offers a new approach to building molecular switches with broad prospects for components and for applications.

In terms of the device application, the cavity array offers the opportunity of introducing pH gradients and bR photoactivity into the device. The fluidity and addressability of the substrate can also in future offer the prospect of building complex membrane switches where initiation of aggregation processes requiring lateral fluidity is enabled.

## Supporting Information Available

bR purity, labeling, proteoliposome preparation, fabrication of PDMS, gold microcavity arrays, and additional data (PDF)
