# Supplementary material for: Robust Photoelectric Biomolecular Switch at a Microcavity-Supported Lipid Bilayer
Source: ACS Appl Mater Interfaces. 2021 Jun 14;13(24):29158–69. doi: 10.1021/acsami.1c06798 (PMC8289237; doi:10.1021/acsami.1c06798)
Supplement: Supplementary file 1 — am1c06798_si_001.pdf [file am1c06798_si_001.pdf]

Supporting Information

A Robust Photoelectric Biomolecular Switch at a Microcavity Supported Lipid Bilayer.

Guilherme B. Berselli, Aurélien V. Gimenez, Alexandra O'Connor and Tia E. Keyes *

School of Chemical Sciences, National Centre for Sensor Research, Dublin City University, Dublin, Ireland

AUTHOR INFORMATION

Corresponding author:

**E-mail: tia.keyes@dcu.ie*

TECHNICAL DATA SHEET

BACTERIORHODOPSIN

Some microorganisms have adapted to the high salinity conditions of this environment, such as the species *Halobacterium salinarum*, from which **Bras del Port** has extracted **Bacteriorhodopsin** in the form of a high quality lyophilized purple membrane.

It is a photosensitive protein with important properties that will improve information storage and processing systems (manufacture of optical processors, 3D memories, biosensors, light energy converters in electric systems, among other uses).

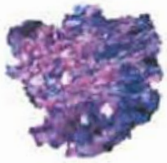

- **Product:** Bacteriorhodopsin in the form of lyophilized *Halobacterium salinarum* MP purple membrane
- **Appearance:** Purple powder
- **Information:**
  - ➤Bacteriorhodopsin MW: 26.5 KD
  - ➤Aminoacids: 248
  - ➤Extinction coefficient: 63,000 M^{-1} cm^{-1}

ANALYSIS* (for a homogenized suspension of 0.5 mg/ml of product in ultra-pure water)

PROTEIC PURITY
> 99% (evaluated in SDS-PAGE)

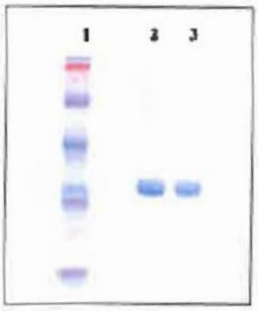

1. MW standard
3. MP-Bras-05

**MAXIMUM ABSORPTION:** 568 nm	A_{280}/A_{570} **RATIO:** 2.0

ABSORPTION SPECTRUM (250-700 nm)

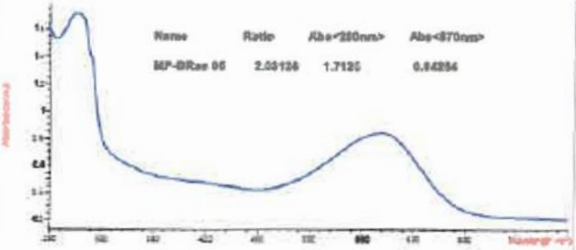

* Analysis January 18th, 2016 (Batch MP-Bras-05)

PACKAGING: 10 mg / 25 mg / 50 mg / 100 mg / 200 mg

CONSERVATION AND STORAGE: Lyophilized powder is very stable for prolonged periods of time being kept dry at room temperature. Aqueous suspensions can be stored at +4ºC for short time, but for longer periods, freezing at -20ºC is recommended.

HANDLING: Purple membrane in suspension tends to keep certain degree of aggregation. Homogenization (e.g. sonication) is recommended to obtain best accuracy.

1st European Maritime Saline certified with the ISO 9001:2008 in all product[illegible].

BRAS DEL PORT, S.A.
SERVICIO DE CALIDAD
31.07.2020

BRAS DEL PORT, S.A.
Ctra. Cartagena-Alicante, km 85 | PO Box, 1 | 03130 SANTA POLA (Alicante)
Tfno. 96 541 33 47 Fax 96 541 55 13 | e-mail salinas@brasdelport.com | www.brasdelport.com

SC-BP-FT-107 ed.1 review 30/07/2020

Labelling of bacteriorhodopsin for Fluorescence Lifetime (Cross)Correlation Spectroscopy - FLCCS/FLCS

The incorporation of bacteriorhodopsin (bR) to proteoliposomes and to MSLBs was conducted using fluorescence studies, such FLCCS and FLCS. To do so, bR was marked ATTO-532 using NHS-ester coupling to an amino residue to covalently bound the ATTO dye to the protein. The labelling procedure used was provided by ATTO-TECH and followed as it is. The labelling efficiency was evaluated by UV-Vis spectroscopy and the degree of labelling (DOL) was measured after dialysis of unreacted ATTO532 dye from the labelled protein. Figure S7 shows the UV-Vis absorption spectra of unlabelled bR (black line) and labelled bR-ATTO532 (green line). The DOL was calculated by comparing the absorbance of protein versus labelled protein using equation S1, indicating a DOL of approximately 60%.

$$DOL = \frac{A_{ATTO532} \cdot \varepsilon_{bR,280}}{(A_{bR,280} - A_{ATTO532}) \cdot \varepsilon_{ATTO532}} \qquad \text{eq. (S1)}$$

Here the extinction coefficient of ATTO532 at 532nm and bR at 280nm were $\varepsilon_{ATTO532}$ = $1.15x10^5$ $M^{-1}cm^{-1}$ ([1]) and ε_{bR280} ≈ 63000 $M^{-1}cm^{-1}$ ([2]), respectively.

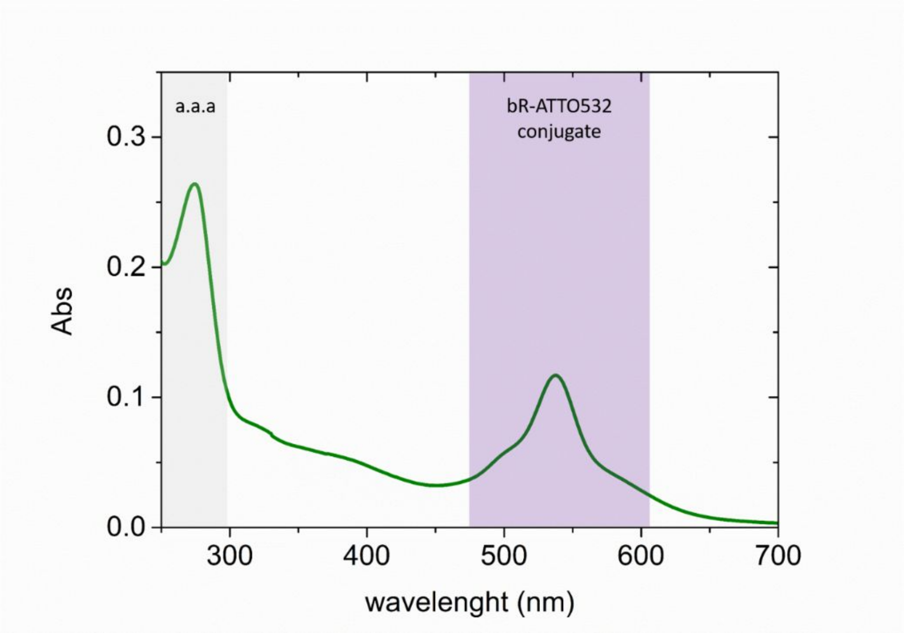

Figure S1. UV-Vis spectra of bR after NHS-ester coupling of bR with ATTO532. The grey insertion represents the aromatic amino acids (a.a.a) absorption region.[3] The purple insertion illustrates the bR-ATTO532 conjugate absorption with maximum absorbance 530-550 nm.

Determination of optimal Titron-X100 concentration for protein incorporation

The optimal concentration of Titron-X100 for the protein incorporation using liposomes comprised of DOPC was determined by the change on the optical density of the liposomes solution, as previously proposed (4). Additionally, the change on the liposomes size was monitored by Dynamic Light Scattering (DLS) (Malvern, Zetasizer). The objective of this method is to create spaces into the liposome with the detergent and fill the gaps with bR. The UV-Vis (Figure S5, open blue circles) shows that the optical density (OD) of the solution against 540 nm initially increases to around 2 mM of Triton X-100. Then OD linearly decreases until approximately 10.5 mM of Triton X-100. At this point, the liposomes are partially to completely dissolved by the detergent in solution.[4] The size of liposomes monitored against detergent concentration indicates that the liposomes swell due to the insertion of detergent to the lipid bilayer until disruption at 10 – 11 mM. (Figure S5, black circles) Therefore, to avoid partial disruption of the liposomes during bR incorporation, the concentration of Triton X-100 used in this work was 5 mM.

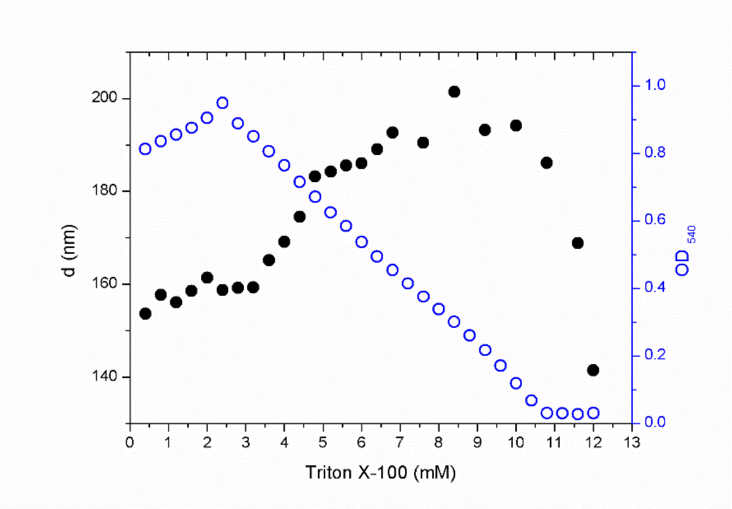

Figure S2. Titration of DOPC liposomes (4mg/ml) against Triton X-100. Blue open circles express the OD collected at 540nm. Full black circles correspond to the size of the liposomes measured by DLS.

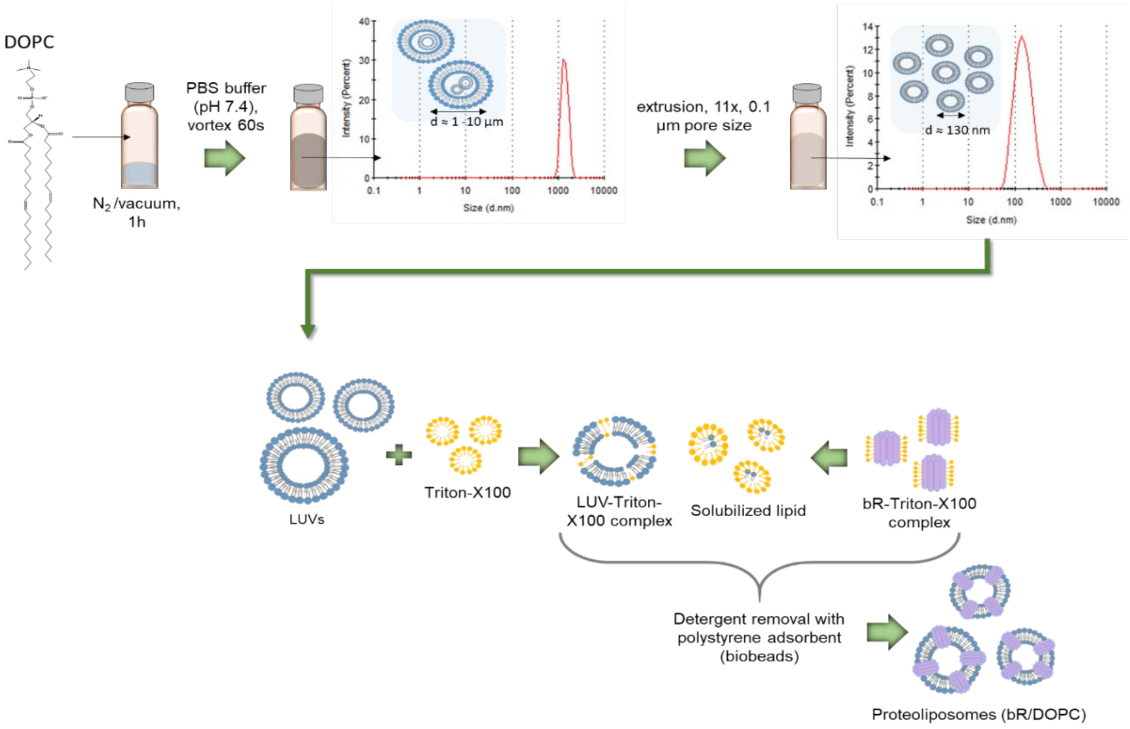

Schematic S1. Schematic illustration of proteoliposomes preparation comprised of DOPC and containing bR.

Electroplating of Gold

In order to obtain a semi-hemispherical gold microcavity array, gold was electrodeposit to pre-casted monolayer of polystyrene microspheres using a cyanide free electroplating solution (Technic Inc.) The gold solution was purged with nitrogen for at least 30 min before reaction. Gold was electrodeposited at -0.6 V (vs. Ag/AgCl) using a 3-electrod set up with the gold array as working electrode, Ag/AgCl (KCl 1M) as reference electrode and a platinum wire as counter-electrode. The gold deposition was controlled by the evolution of current over time (Figure S3a). The electrodeposition was stopped at the lower current, which represents when metal had grown to the hemispherical point of the PS spheres. To remove residual electroplating gold solution, the resulting substrate is rinsed with deionized water, ethanol and dried under nitrogen flow. Then, the substrates were electrochemically cleaned in H_2SO_4 (10mM) through cyclic voltammetry (Figure S3b). The substrates were then submerged in 1 mM 6-mercapto-1-hexanol in ethanol to allow self-assembled monolayer formation (SAM) overnight (Figure S3c). After SAM formation the PS spheres were removed by sonicating the substrates in THF for 5 min. To prefill the microcavities with the conducting electrolyte the substrates were sonicated in 0.1 M KCl for 10 minutes prior to lipid bilayer assembly.

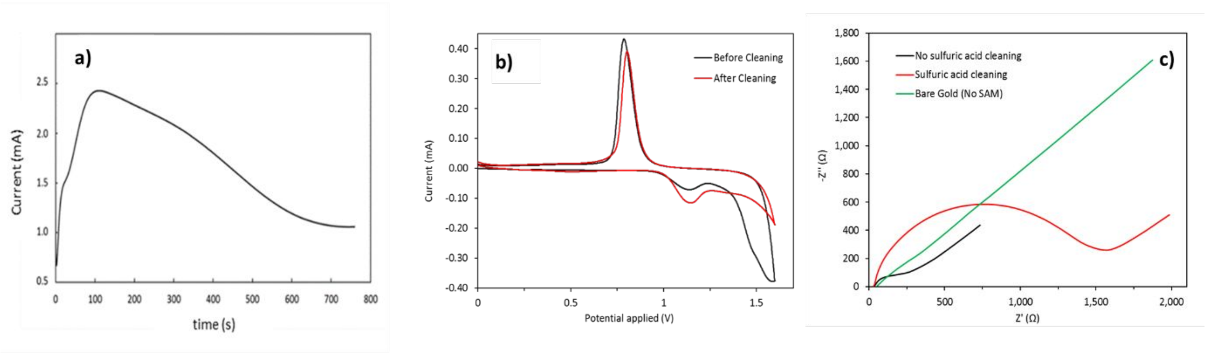

Figure S3. (a) Time-current of gold electroplating. (b) Sulphuric acid potential cycling cleaning of gold microcavity arrays before (black line) and after (red line) 6 cycles at 40 mV/s versus (Ag/AgCl) reference electrode. (c) Shows the Nyquist plots obtained after formation of self-assembled monolayer (SAM) of 6-mercapto-1-hexanol over gold-microcavity arrays before (black line) and after (red line) potential cycling cleaning. Green line represents the impedance spectroscopy of a bare gold substrate without SAM. The impedance was evaluated

in 1 mM of ferricianide/ferrocyanide ($Fe^{2+}/^{3+}$) with 0.1M KCl at 0.26V against (Ag/AgCl) reference electrode. The frequency range of measurement was 10^4 Hz – 10^{-1} Hz.

Characterization of PDMS and Gold µcavity arrays

The lipid bilayers comprised of DOPC/bR were spanned across PDMS and Gold arrays prepared using polystyrene (PS) spheres templating. In order to obtain a highly closed packed array, the Gold arrays were prepared by gravity-assisted lithography of PS, followed by gold electroplating. The dimensions of microcavities were analyzed with FESEM as illustrated in Figures S4. Top line shows the microcavity arrays obtained after gold electroplating and PS removal, indicating the highly ordered closed packed arrays over micrometer range. In order to highlight the lateral structure and the individual cavities dimensions on gold-arrays, tilted (+25°) and profile images were collected (bottom line), indicating depth and diameter of approximately 0.5 µm and 1 µm, respectively.

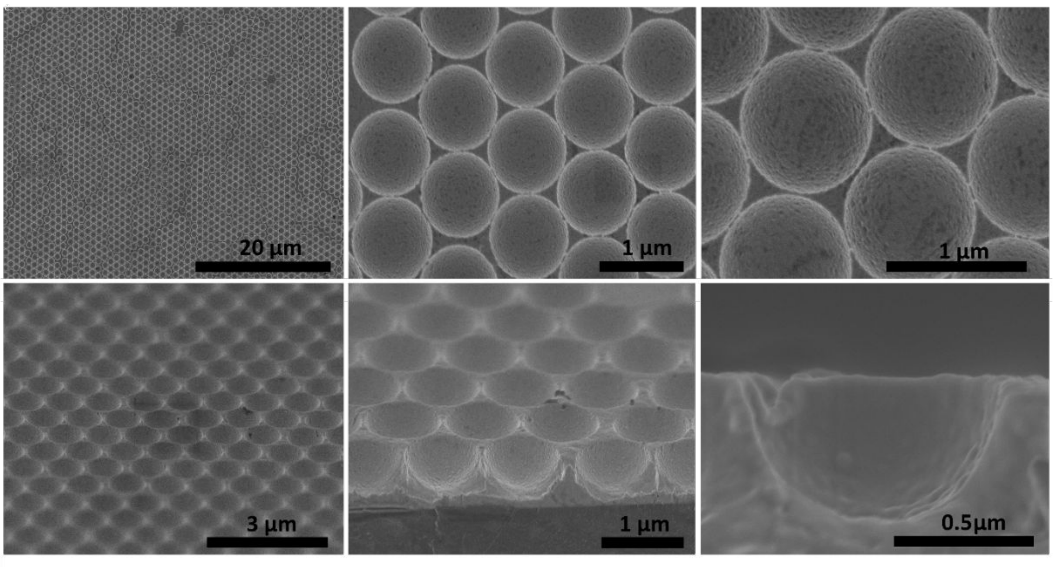

Figure S4. Field Emission Scanning Electron Microscopy (FESEM) of gold microcavity array formed by gold electrodeposition to pre-casted PS microspheres (1 um) monolayer. The top

line shows the hexagonal highly ordered array from topview. The tilted and profile images in the bottom line illustrates the lateral and axial dimensions obtained for gold microcavities to be approximately height = 0.50 µm and diameter = 1 µm.

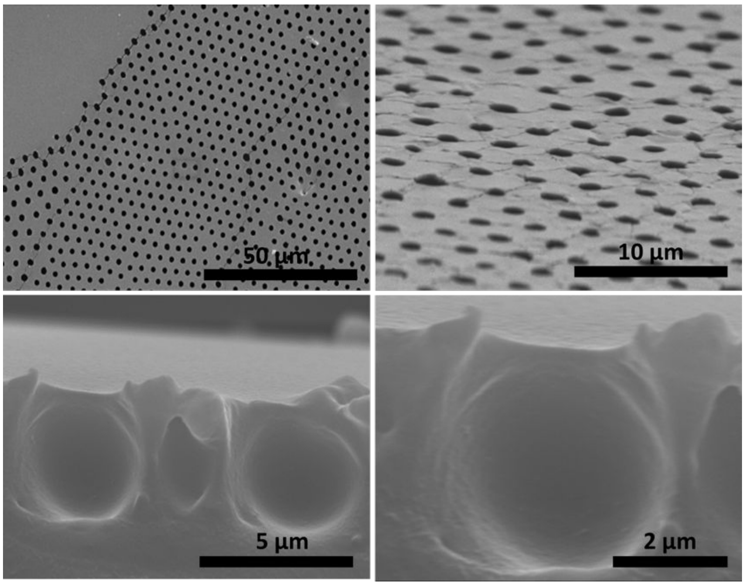

Figure S5. Scanning electron microscopy (SEM) of PDMS microcavity arrays. Top shows the microcavity array after PS removal over a large area and a tilted close to the array, respectively. Figures (c) and (d) show the profile structure of individual microcavities. The dimensions obtained for the microcavities were approximately height = 3.2 µm, diameter = 4.5 µm.

Electrochemical Impedance Spectroscopy and Chronoamperometry of DOPC lipid bilayers

Figure S6a shows the Nyquist plot obtained after incorporation of bR to MSLBs. The impedance was evaluated in 0.1 M KCl as supportting electrolyte (pH 5.5) at 0V (vs Ag/AgCl) using an AC amplitude of 0.01 V with a frequency range of 10^4 Hz to 10^{-1} Hz. The membrane resistivity (Rm) and capacitance (Cm) obtained for the lipid membranes after fitting to model previously reported are displayed in table S1. The membrane resistance and capacitance are obtained from Rm and Cm as approximatley 6 MOhm and 6.1 µF.Sm-1 in absence of protein

which is in good agreement with previous values reported by our group[5] and similar though somewhat lower and higher respectively than reported for tethered supported lipid bilayers at smooth gold surface. The difference reflects the fact that support is porous and the gold cavity interior umodified. SAM is only applied to the top exterior surface of the array and bilayer spanning the pores without underlying SAM.[6]

Table S1. Electrochemical properties of lipid bilayers containing bR.

bR (mol %)	Rm (MΩ)	Cm (μF.S^{m-1})
0.01	4.8 ± 0.8	3.1 ± 0.1
0.03	3.7 ± 0.5	4.5 ± 0.1
0.3	2.2 ± 0.4	6.3 ± 0.2
3	1.2 ± 0.2	8.7 ± 0.3

Figure S6b shows the photocurrent response of a DOPC bilayer in absence of bR to the 2 mW LED at wavelength 550 nm. Here, a sharp photo-current signal of approximatly 7 nA/cm^2 was observed with LED switched on. This small contribution to a change in current may be an artifact of the photoelectric effect or due to static interference to the electronic aparatus.

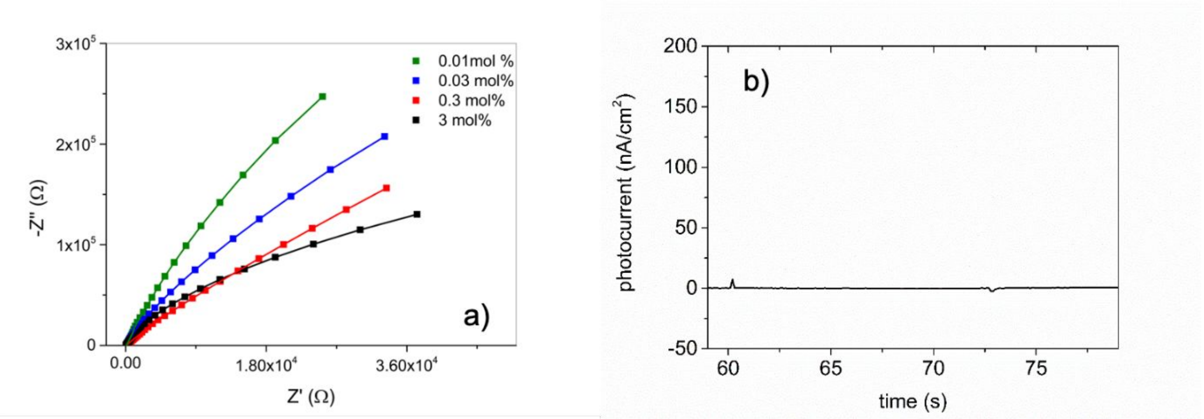

Figure S6. (a) EIS of MSLBs containing bR. (b) Photo-current of a gold-MSLB comprised of DOPC, i.e. background photocurrent in absence of reconstituted bR.

Table S2. Fitted decays of bR at different concentrations. A represents the amplitude and t decay time.

Concentration of bR (mol%)	Photoactivation				Photodeactivation	
	I_{fast} (nA/cm2)	τ_{fast} (s)	I_{slow} (nA/cm2)	τ_{slow} (s)	I (nA/cm2)	τ (s)
3	208.3 ± 31.2	0.037 ± 0.06	23.7 ± 5.4	0.365 ± 0.025	-133.5 ± 15.2	0.155 ± 0.057
0.3	88.9 ± 15.4	0.026 ± 0.04	5.67 ± 1.6	0.276 ± 0.024	-47.5 ± 10.7	0.122 ± 0.026
0.03	29.3 ± 7.5	0.031 ± 0.03	2.73 ± 0.4	0.321 ± 0.031	-23.2 ± 6.3	0.134 ± 0.018
0.01	4.37 ± 1.2	0.021 ± 0.02	0.47 ± 0.02	0.252 ± 0.012	-13.1 ± 4.2	0.112 ± 0.027

Table S3. Kinetics of photoactivation of bR-MSLBs containing 3 mol% of protein within a range of pH (3.5 – 9.5).

pH	Photoactivation (ON)			
	I_{fast} (nA/cm2)	t_{fast} (s)	I_{slow} (nA/cm2)	t_{slow} (s)
3.5	131.8 ± 27.2	0.021 ± 0.003	5.2 ± 1.6	0.178 ± 0.021
4.5	133.5 ± 25.4	0.029 ± 0.006	12.1 ± 3.3	0.192 ± 0.018
5.5	153 ± 31.7	0.027 ± 0.005	7.8 ± 2.5	0.272 ± 0.022
6.5	22.4 ± 9.8	0.031 ± 0.006	9.3 ± 2.2	0.306 ± 0.036
7.5	17.1 ± 3.6	0.029 ± 0.004	6.2 ± 1.5	0.421 ± 0.052
8.5	32.1 ± 7.5	0.032 ± 0.007	2.6 ± 1.1	0.698 ± 0.041
9.5	62.9 ± 13.7	0.029 ± 0.005	1.2 ± 0.8	0.783 ± 0.083

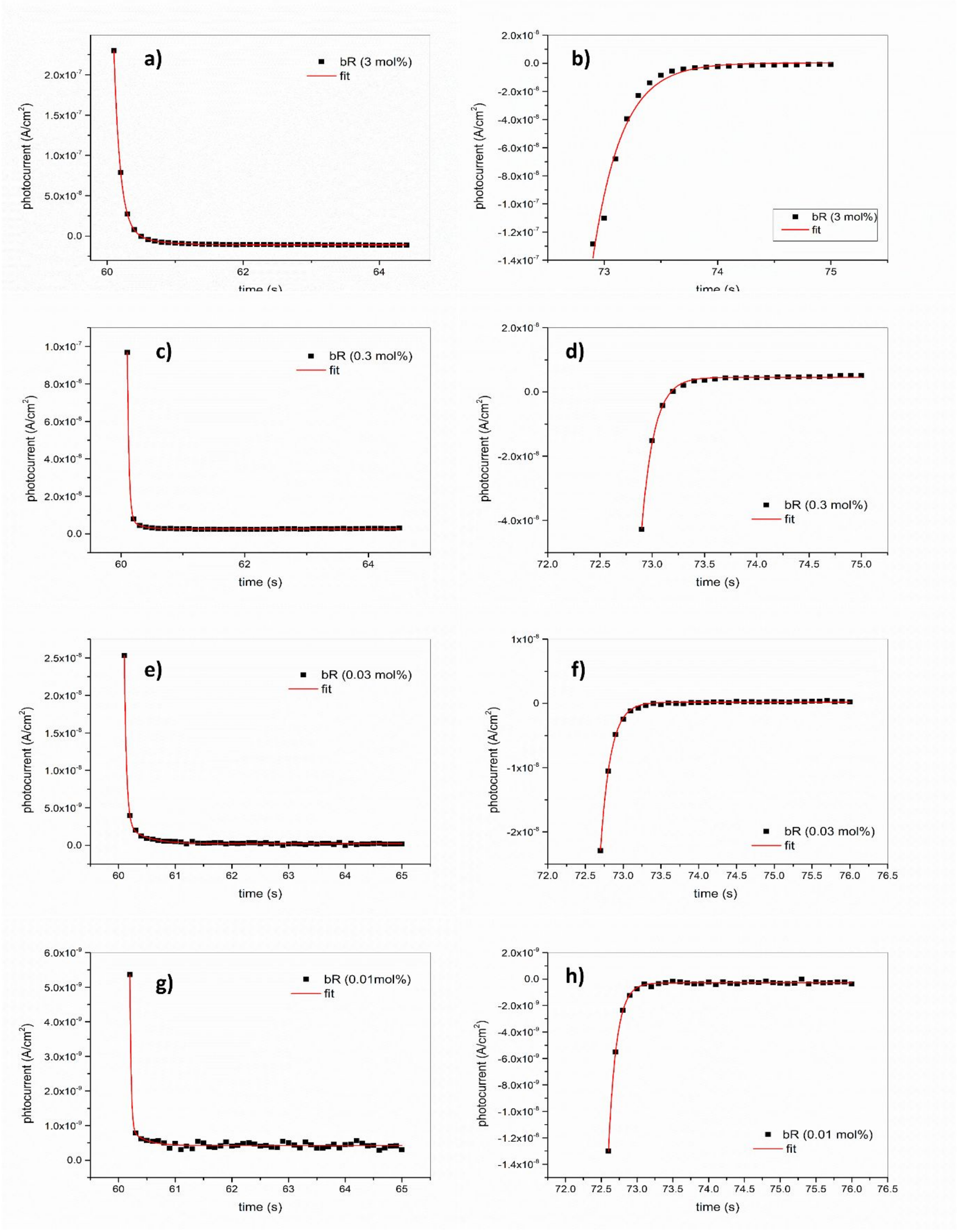

Figure S7. Fitted photocurrent decays of bR-MSLBs. (a) and (b), (c) and (d), (e) and (f), (g) and (h) show the fitted decays of activation and deactivation of bR-MSLBs containing 3 mol%, 0.3 mol%, 0.03 mol% and 0.01 mol% respectively.

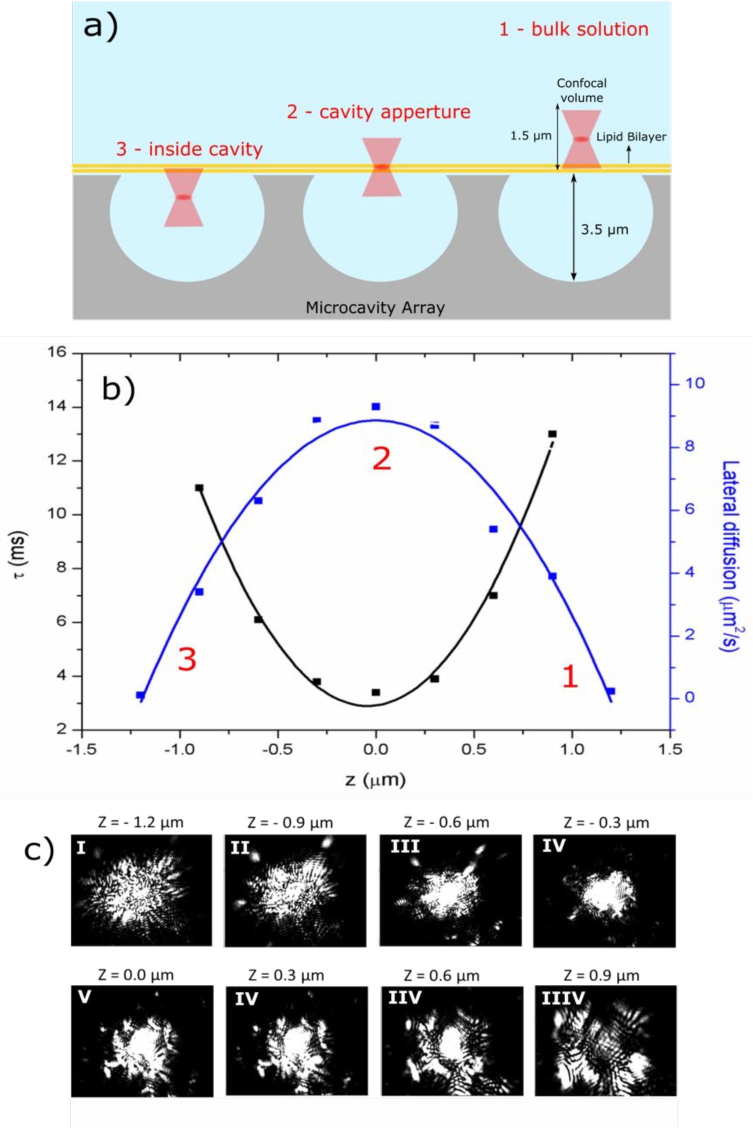

Figure S8. Z-scan obtained for Microcavity Supported Lipid Bilayers at a single cavity. a) Schematic representation of the position of the confocal volume within the substrate. b) Indicates the lateral diffusion of labelled DOPE-ATTO655 within the focal plan of the sample. c) Actual image obtained from the focal spot using a CCD camera.

Membrane impermeable probe pyranine was introduced in the pores of the array before assembly of the bilayer presonicating the microcavity arrays in 1 µM pyranine solution. After, we prepared a lipid bilayer using the method here, by LB followed by proteoliposomes fusion.

The substrate was imaged using a confocal microscope (Leica TCS SP8, Ex: 405 nm, detection window 440 - 470nm). As shown in figure S9, we observed that after the bilayer preparation, the dye remain encapsulated within the microcavities, which indicate the bilayer is both suspended across the pore aperture and forms an effective impermeable seal, this persists in this way for several hours up to days

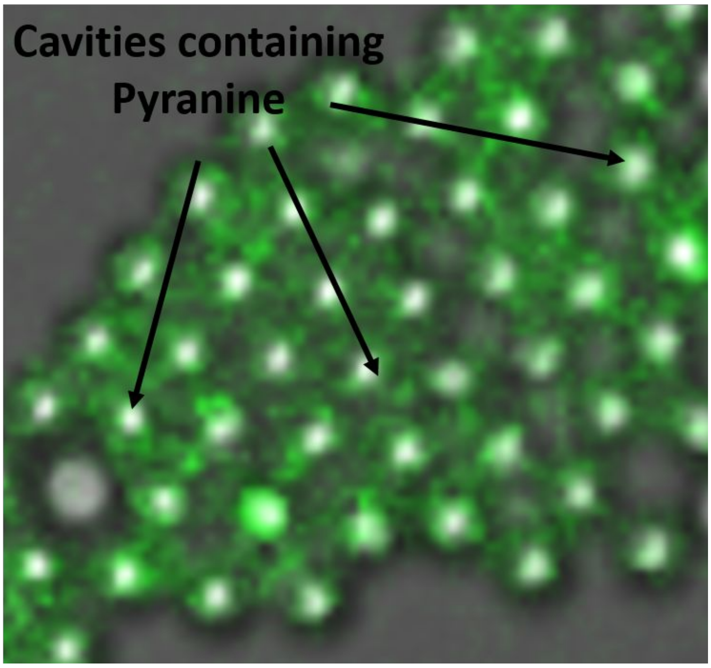

Figure S9. Membrane Leakage test using membrane impermeable probe pyranine. The pryanine is introduced by sonication in solution into the microcavities by prior to monolayer transfer and proteoliposomes fusion. We observed that once the bilayer is in place the pyranine was retained within the cavity with no loss of emission intensity over the window of our experiments confirming the bilayer was spanning and its integrity maintained during our experimental windows.

References

(1) Hughes, L. D.; Rawle, R. J.; Boxer, S. G. Choose Your Label Wisely: Water-Soluble Fluorophores Often Interact with Lipid Bilayers. *PLoS One* **2014**, *9* (2). https://doi.org/10.1371/journal.pone.0087649.

(2) London, E.; Khorana, H. G. Denaturation and Renaturation of Bacteriorhodopsin in Detergents and Lipid-Detergent Mixtures. *J. Biol. Chem.* **1982**, *257* (12), 7003–7011.

(3) Antosiewicz, J. M.; Shugar, D. UV–Vis Spectroscopy of Tyrosine Side-Groups in Studies of Protein Structure. Part 2: Selected Applications. *Biophys Rev* **2016**, *8* (2), 163–177. https://doi.org/10.1007/s12551-016-0197-7.

(4) Geertsma, E. R.; Nik Mahmood, N. A. B.; Schuurman-Wolters, G. K.; Poolman, B. Membrane Reconstitution of ABC Transporters and Assays of Translocator Function. *Nature Protocols* **2008**, *3* (2), 256–266. https://doi.org/10.1038/nprot.2007.519.

(5) Sarangi, N. K.; Stalcup, A.; Keyes, T. E. The Impact of Membrane Composition and Co-Drug Synergistic Effects on Vancomycin Association with Model Membranes from Electrochemical Impedance Spectroscopy. *ChemElectroChem* **2020**, *7* (22), 4507–4507. https://doi.org/10.1002/celc.202001281.

(6) Basit, H.; Van der Heyden, A.; Gondran, C.; Nysten, B.; Dumy, P.; Labbé, P. Tethered Bilayer Lipid Membranes on Mixed Self-Assembled Monolayers of a Novel Anchoring Thiol: Impact of the Anchoring Thiol Density on Bilayer Formation. *Langmuir* **2011**, *27* (23), 14317–14328. https://doi.org/10.1021/la202847r.
